# The Application of Self-Healing Microcapsule Technology in the Field of Cement-Based Materials: A Review and Prospect

**DOI:** 10.3390/polym15122718

**Published:** 2023-06-17

**Authors:** Bo Liu, Mingli Wu, Wei Du, Lu Jiang, Hongjun Li, Luoxin Wang, Jinhui Li, Danying Zuo, Qingjun Ding

**Affiliations:** 1 School of Materials Science and Engineering, Wuhan Textile University, Wuhan 430200, China; liubo13006311228@163.com (B.L.); jl0107023@163.com (L.J.); wanglx@wtu.edu.cn (L.W.); jhli@wtu.edu.cn (J.L.); 2 College of Life Science & Technology, Huazhong University of Science and Technology, Wuhan 430074, China; d202280823@hust.edu.cn; 3 State Key Laboratory of Silicate Materials for Architectures, Wuhan University of Technology, Wuhan 430070, China; 4 Hubei Provincial Engineering Research Center of Industrial Detonator Intelligent Assembly, Wuhan Textile University, Wuhan 430073, China; lhj@wtu.edu.cn

**Keywords:** microcapsule, self-healing, structures of cement-based materials, physical trigger, chemical trigger, electromagnetic-trigger

## Abstract

This review provides an overview of microcapsule self-healing technology and its application in the field of cement-based materials, as well as future prospects. The presence of cracks and damage in cement-based structures during service has a significant impact on their lifespan and safety performance. Microcapsule self-healing technology shows promise in achieving self-healing by encapsulating healing agents within microcapsules, which are released upon damage to the cement-based material. The review starts by explaining the fundamental principles of microcapsule self-healing technology and explores various methods for preparing and characterizing microcapsules. It also investigates the influence of incorporating microcapsules on the initial properties of cement-based materials. Additionally, the self-healing mechanisms and effectiveness of microcapsules are summarized. Finally, the review discusses the future development directions for microcapsule self-healing technology, outlining potential areas for further research and advancement.

## 1. Introduction

**Figure 1:**
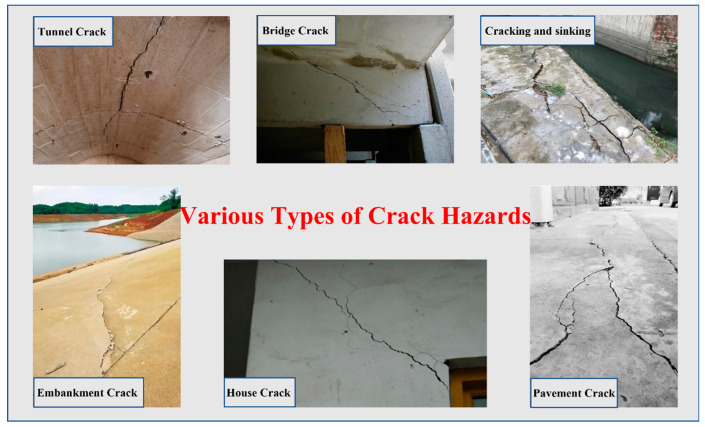
Several typical forms of crack damage in cement-based materials structures.

The cement-based material is a composite material made up of cement, coarse and fine aggregates, water, and additives. Due to its advantages of low raw material cost, high compressive strength, and ease of processing and shaping, cement-based materials have been widely used in engineering construction such as buildings, bridges, tunnels, and port embankments [1,2,3,4]. However, due to its inherent porous and brittle nature, cement-based materials are prone to cracking [5,6,7,8,9,10]. Cracks not only reduce the strength of cement-based materials, but also provide pathways for harmful ions, acidic media, and other substances to penetrate the interior of cement-based materials, making it vulnerable to corrosion and further exacerbating the corrosion of internal steel reinforcement, which poses a great threat to structures of cement-based materials [11,12]. If cracks are not healed promptly and effectively, they not only reduce the durability of cement-based materials structures, but also may trigger macro-cracks from microscopic ones, ultimately leading to brittle fracture, resulting in safety accidents and significant economic losses [13,14]. Therefore, timely crack heal is crucial for prolonging the service life of cement-based materials structures. This article aims to present the current state of knowledge on the subject of crack self-heal in cement-based materials [15,16,17]. Several typical forms of crack damage in structures of cement-based materials are shown in Figure 1.

To improve the service life of cement-based materials, countries around the world invest significant amounts of manpower, financial resources, and materials each year to heal cement-based materials cracks and damage [18]. Traditional methods for healing cement-based materials cracks include surface coatings, grouting, groove filling, and structural strengthening [19,20,21,22,23]. Surface coating is the most common method for crack heal, also known as surface sealing or application sealing. This method involves applying a coating to the surface layer of cement-based materials to heal micro-cracks with a width less than 0.2 mm. It can also be used for the decoration and waterproofing of the cement-based materials surface, as well as to prevent carbonation and harmful ion corrosion. Grouting involves using a specific pressurized conveying device to apply 0.2~0.4 MPa pressure to the bonding material, which is then forced into the cement-based materials cracks. After the bonding material hardens at the crack, it becomes one with the matrix to achieve filling, sealing, and reinforcement of the crack. This method is widely used and applicable to cracks of all sizes, from micro-cracks to macro-cracks that affect the overall integrity of the structure and have certain waterproofing effects. Groove filling is used to directly fill cracks with healing materials when the crack width is less than 0.3 mm or the depth is shallow, or when grouting cannot be fully healed. The specific implementation method is as follows: first, chisel a V-shaped, trapezoidal, or U-shaped groove along the crack, with the depth and width of the groove controlled at approximately 40–60 mm; second, remove residue from the groove with compressed air and then rinse with high-pressure water; third, use plastic or rigid waterproof material to fill the groove to achieve crack sealing. Although this method can heal cracks visible on the surface of components, it cannot heal internal cracks in the filled cement-based materials, and micro-cracks will still remain inside the structure, which has a negative impact on safe use of the components. Structural strengthening is a heal method for cracks that affect structures of cement-based materials. For cracks that affect cement-based materials building structure characteristics, reinforcement methods need to be considered. To prevent the continued extension of wide and unstable cracks in the development stage, cross-crack nails can be used for control. However, these traditional heal methods generally have high maintenance costs, cannot effectively heal internal cracks, and some healed cracks are prone to secondary cracking, limiting their effectiveness in extending the service life of cement-based materials [24].

Considering this, it is imperative to urgently develop cement-based materials that possess self-healing capabilities. This would effectively enhance the durability of such materials and improve their safety during usage. Recently, with the introduction of new building materials and the concept of intelligent cement-based materials, researchers have carried out research on intelligent cement-based materials with self-healing capabilities to address cement-based materials crack problems [25,26,27,28]. Based on the principle of self-healing in living organisms, cement-based materials crack self-healing occurs when the intrinsic structure of cement-based materials is stimulated by external stimuli or when self-healing materials are added to the cement-based material, allowing the cement-based material to spontaneously heal cracks [29,30]. Existing cement-based materials self-healing methods include intrinsic self-healing, crystalline self-healing, microbial self-healing, hollow fiber self-healing, and microcapsule self-healing using stored healing agents [31,32,33,34,35]. Among these, microcapsules self-healing has received extensive research attention from domestic and foreign scholars, as they are the most promising self-healing materials for engineering applications [36,37]. The spherical structure of microcapsules allows them to come into full contact with tiny cracks from all directions, increasing the triggering rate and healing cement-based materials. Additionally, microcapsules of different particle sizes can be prepared by changing the preparation conditions to meet the heal requirements of materials with different pore structures [38,39].

In recent years, research on the self-healing properties of cement-based materials doped with microcapsules has received widespread attention, mainly including the following aspects: (1) preparation methods of microcapsules; (2) intrinsic properties of microcapsules; (3) the effect of microcapsule addition on the initial properties of the cement-based material; and (4) the self-healing mechanism and effect of microcapsules. Based on existing research, this article mainly summarizes and analyzes the achievements in the above aspects and discusses future directions for further research in this field, in order to provide reference for related research on microcapsule self-healing of cement-based materials.

## 2. Preparation Methods of Microcapsules

Currently, there are over 200 types of microcapsule preparation techniques, which can be mainly classified into chemical, physical, and physicochemical methods based on the different mechanisms of capsule shell formation. Although there are many methods for preparing microcapsules, the preparation methods for self-healing microcapsules used to encapsulate healing agents are mainly interfacial polymerization method, in situ polymerization, spray drying method, and melt condensation method, according to research cited in the scientific literature.

### 2.1. Interfacial Polymerization Method

The interfacial polymerization method was initially invented by DuPont Company for preparing copolymers containing two monomers. This method involves dissolving two monomers containing multiple functional groups in a solution that is immiscible, and conducting the polymerization reaction at the interface of the two phases to form the polymer. When preparing microcapsules using the interfacial polymerization method, water and organic solvents are commonly chosen as the liquid phase system. During the reaction, the two monomers are dissolved separately, and the core material is dissolved in the dispersed phase. If the core material is soluble in water, an oil-in-water emulsion is formed, whereas if it is soluble in organic solvents, a water-in-oil emulsion is formed. Emulsifiers are added to stabilize the system. During the reaction process, the monomers in both phases diffuse to the interface of the emulsion, where they undergo polymerization to form microcapsules that encapsulate the core material [40]. Yang et al. [41] used the interfacial polymerization method to prepare polymer-based self-healing microcapsules using polyurethane as the shell material and IPDI as the core material.

### 2.2. In Situ Polymerization

In contrast to the interfacial polymerization method, which involves the reaction between two immiscible monomers at the phase boundary, the in situ polymerization method utilizes soluble monomers (oligomers) and initiators, while the resulting polymer becomes insoluble and precipitates from the solution during the later stages of the reaction. In the preparation of microcapsules using in situ polymerization, the monomers (oligomers) and initiators are added to either the dispersed or continuous phase. Initially, the monomers form low molecular weight prepolymers, which gradually deposit on the surface of the core material as the molecular weight of the prepolymer increases. As the polymerization reaction proceeds, these prepolymers undergo cross-linking to form macromolecules, creating a shell that encapsulates the core material. The preparation process of microcapsules through in situ polymerization is relatively straightforward, with a crucial step being the control of polymer deposition on the core material’s surface to achieve shell formation [42]. Currently, the most commonly employed method for producing self-healing microcapsules is in situ polymerization, with urea-formaldehyde resin being frequently used as the material for the capsule shell [43].

### 2.3. Spray Drying Method

Spray drying is a method of drying solid solutions by using hot air to quickly evaporate the water content, resulting in a solid powder. This method is commonly used in the preparation of powdered milk, coffee, and laundry detergent. When used to prepare microcapsules, the first step is to prepare a solution of capsule shell material to enclose the core material in an emulsion dispersion. Then, a spray device is used to disperse the solution into uniform spherical droplets. Under the action of hot air, the water in the capsule shell solution evaporates, causing the encapsulated core material to form into capsule particles. This method for preparing microcapsules is fast and simple, but defects in the capsule shell can easily lead to leakage of the core material [44]. Liu et al. [45] employed the spray drying technique to fabricate self-healing microcapsules. Ethyl cellulose was utilized as the shell material, while sodium monofluorophosphate was selected as the core material. When the matrix is invaded by harmful ions, the shell material of the microcapsules undergoes degradation, leading to the release of the core material and facilitating the healing process.

### 2.4. Melt Condensation Method

The melt condensation method is a purely physical method. Firstly, the raw material of the shell is melted, and the core material is uniformly dispersed in this liquid shell material. Then, an inert liquid medium is added to the mixture, causing the shell material to solidify and encapsulate the core material to form microcapsules. The prerequisite for using this method is that the core material, shell material, and low-temperature liquid medium are all insoluble, and can stably exist within the temperature range of the melted shell material. The key to forming microcapsules is to ensure that the shell material can provide good coverage of the core material and prevent separation of the two during the cooling process. Wetting dispersants can be added during the core material dispersion process to increase the stirring speed, and the viscosity and temperature of the dispersion system should be controlled to avoid this situation from occurring [46]. Du et al. [46] used paraffin as the shell material and toluene diisocyanate (TDI) with high reactivity and extremely low viscosity as the core material to prepare microcapsules for self-healing of concrete. TDI reacts with the water can generate polyurea quickly for self-healing of the crack.

## 3. Intrinsic Properties of Microcapsules

### 3.1. Core Fraction

Li et al. [47] prepared microwave-triggered burst release microcapsules containing TDI by encapsulating it with a mixture of paraffin, polyethylene wax, and graphene as the shell material. When the mass ratio of paraffin to polyethylene wax was changed from 1:1 to 4:1, the core fraction of the microcapsules increased from 69.3% to 73.4%. This was because the mixture with less polyethylene wax cooled faster during the encapsulation process, thereby encapsulating more TDI in the microcapsules. When the mass ratio of paraffin to polyethylene wax was 1:4, the cooling rate of the shell mixture was too slow to form microcapsules upon addition of perfluorotributylamine. Therefore, the mass ratio of paraffin to polyethylene wax of 4:1 was the suitable condition for obtaining high core fraction microcapsules.

**Figure 2:**
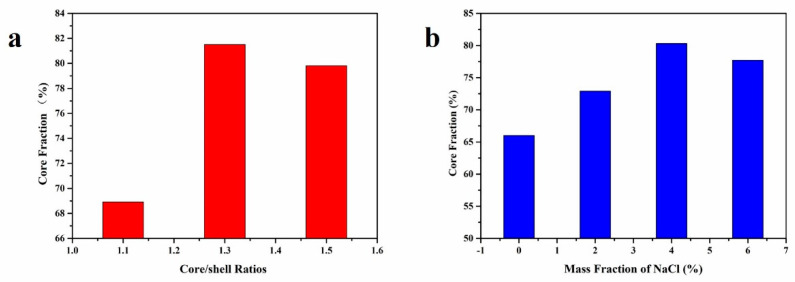
(**a**) Core fraction of microcapsules with different core/shell ratios, (**b**) Effect of NaCl on core fraction of microcapsule.

In a study conducted by Xue et al. [48], microcapsules were synthesized using the in-situ polymerization method, with urea-formaldehyde resin as the shell material and rejuvenator as the core material. The investigation revealed that the core fraction of the microcapsules initially increased and then decreased as the core-shell ratio was varied. The highest core fraction was observed at a core-shell ratio of 1.3:1 (Figure 2a). Excessive shell material led to prepolymers’ aggregation, resulting in a reduced coating efficiency. On the other hand, insufficient shell material failed to adequately cover the rejuvenator droplets, and thin shells were susceptible to shear-induced damage, resulting in a lower core fraction. Additionally, the incorporation of NaCl was found to have a significant positive effect on the core fraction of the microcapsules. When the mass fraction of NaCl was set at 4%, the core fraction reached its maximum value of 80.31%. NaCl solution, possessing strong electrolytic properties, increased the ion strength within the system and disrupted the double electric layer’s structure (Figure 2b). As a result, the collisions between prepolymers were enhanced, facilitating the formation of microcapsules.

In a study conducted by Tao et al. [49], microcapsules were fabricated using melamine-formaldehyde resin as the shell material and shellac as the core material. The researchers investigated the effects of four factors on the overall performance of the microcapsules, including the hydrophilic–lipophilic balance (HLB) value of the emulsifier, solvent type, mass ratio of shellac to rosin, and rotation speed. The results highlighted the significant influence of the emulsifier’s HLB value as a key factor. To further analyze the relationship between the emulsifier’s HLB value and the morphology and encapsulation efficiency of the microcapsules, and to optimize their performance, a single-factor experiment was conducted with the emulsifier’s HLB value as the sole variable. The findings revealed that when the HLB value was set to 12.56, the microcapsules, consisting of a melamine–formaldehyde resin-coated shellac–rosin mixture, exhibited a uniform distribution and high core fraction.

**Figure 3:**
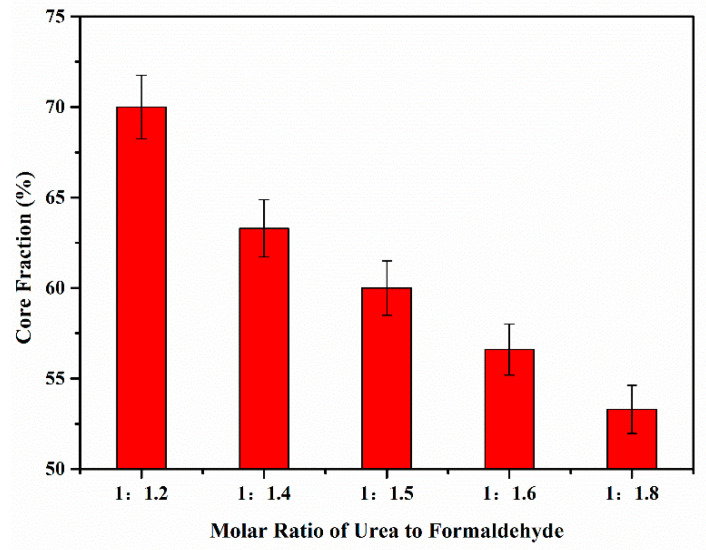
Core fraction of microcapsules.

In a study conducted by Zhao et al. [50], thermochromic microcapsules were prepared using thermochromic compounds as the core material and urea formaldehyde as the shell material. The research findings demonstrated that the molar ratio of urea to formaldehyde significantly influenced the core fraction of the microcapsules (Figure 3). The highest core fraction, measuring at 70.0 ± 1.75%, was achieved at a molar ratio of urea to formaldehyde of 1:1.2. Conversely, the lowest core fraction, measuring at 53.3 ± 1.33%, was observed when the molar ratio of urea to formaldehyde was 1:8. This discrepancy can be attributed to the excessive presence of formaldehyde, resulting in a higher proportion of unreacted hydroxymethyl hydrophilic groups in the condensate. The incomplete condensation adversely affects the core fraction of the microcapsules.

Microcapsules are tiny particles in which a core material is encapsulated within an external shell. The core fraction, which refers to the proportion of the core material covered by the capsule shell, is a crucial parameter that significantly influences the stability, release performance, and application effectiveness of microcapsules. Currently, there are several factors affecting the core fraction of microcapsules. Different healing agents possess distinct surface properties and encapsulation mechanisms, and the core fraction can be controlled by adjusting the concentration, molecular weight, and solution conditions of these agents. Commonly used healing agents include polymers, lipids, and natural polymers. Optimizing the encapsulation process is also an important approach to improving the core fraction. Factors such as capsule formation methods, encapsulation rate, and stirring conditions can affect the core fraction. Researchers can optimize the process parameters, such as pH value, temperature, and stirring speed, to enhance the core fraction. In summary, microcapsules synthesized using various preparation methods and materials can achieve a core fraction of over 70%, ensuring an adequate amount of healing agent for crack self-heal within the microcapsules.

### 3.2. Sealing Performance

**Figure 4:**
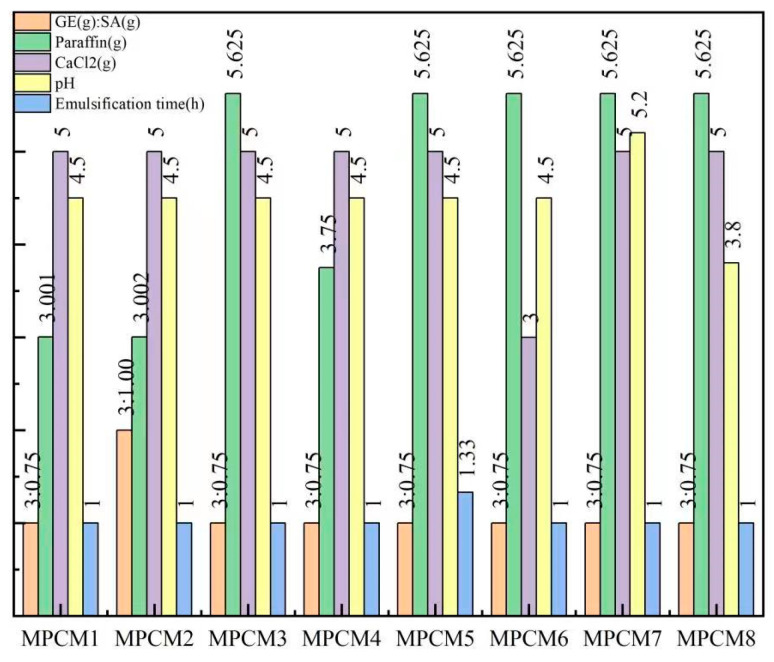
The experimental conditions for preparing MPCMs.

Tian et al. [51] utilized a composite material comprising gelatin and sodium alginate as the shell material and paraffin as the core material to fabricate microcapsules through complex coacervation. The compactness of the microcapsules was evaluated by measuring the leakage of the composite shell material using filter paper. The results demonstrated that the highest compactness was achieved when the leakage of the microcapsules was minimal. Figure 4 illustrates that MPCM5 and MPCM8 exhibited the highest leakage, indicating inadequate coverage of the paraffin by the shell material. On the other hand, MPCM1, MPCM4, MPCM6, and MPCM7 showed slight paraffin leakage, suggesting that a majority of the paraffin was effectively coated by gelatin and sodium alginate. MPCM2 and MPCM3 displayed minimal or no leakage, indicating excellent compactness of the microcapsules.

**Figure 5:**
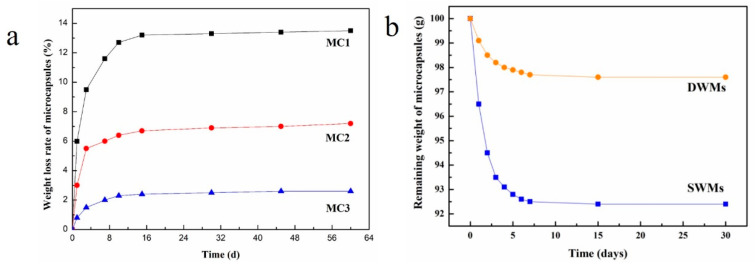
(**a**) Weight loss rates of MC1, MC2, MC3 [52], Copyright, 2020, Elsevier, License Number 5550140573436; (**b**) Remaining weight of SWMs and DWMs [53].

We prepared three types of microcapsules coated with paraffin, paraffin/polyethylene wax, and nano-SiO_2_/paraffin/polyethylene wax, respectively, to encapsulate TDI [52]. The microcapsules were weighed and stored in a constant temperature and humidity chamber at 25 °C and 50% relative humidity. To investigate the compactness of the microcapsules, the weight change in the microcapsules was monitored at 1, 3, 7, 10, 15, 30, 45, and 60 days, respectively. As shown in Figure 5a, the weight loss rates of the microcapsules increased rapidly from 1 day to 10 days and then stabilized after 10 days, remaining relatively constant thereafter. After 60 days of storage, the weight loss rates of paraffin-coated TDI microcapsules (MC1) and paraffin/polyethylene wax composite-coated TDI microcapsules (MC2) were 13.5% and 7.2%, respectively, indicating that the compactness of the paraffin/polyethylene wax composite coating was better than that of the paraffin coating. This is because compared with paraffin, polyethylene wax has a larger molecular weight and density, stronger intermolecular forces, smaller gaps between molecular chain segments, and higher crystallinity, thus improving the compactness of the microcapsule shell material. The weight loss rates of the nano-SiO_2_/paraffin/polyethylene wax composite-coated TDI microcapsules (MC3) was only 2.6% after 60 days of storage, which was attributed to the addition of nano-SiO_2_ to the paraffin/polyethylene wax mixture, which can reduce the micro-defects in the composite coating and make the shell structure more compact, thus avoiding TDI leakage and improving the compactness of the microcapsules.

Based on our previous research, two types of microcapsules were prepared using paraffin and nano-Fe_3_O_4_/paraffin coating on epoxy resin [53]. A total of 100 g of these microcapsules were weighed and placed in an oven (20 ± 2 °C, 50% RH) for varying durations of 1, 2, 3, 4, 5, 6, 7, 15, and 30 days. The compactness of the microcapsules was assessed by measuring the remaining weight of microcapsules at each time point. As shown in Figure 5b, from day 1 to day 7, the remaining weight of both the paraffin-coated epoxy resin microcapsules (SWMs) and the nano-Fe_3_O_4_/paraffin-coated epoxy resin microcapsules (DWMs) decreased significantly and then stabilized, with no further changes in weight after 15 days. After 30 days, the remaining weight of SWMs decreased by 7.6% from the initial value to 92.4 g, while the remaining weight of DWMs decreased by only 2.4% to 97.6 g. This difference can be attributed to the inclusion of nano-Fe_3_O_4_ in the paraffin, which filled micro-defects on the surface of the paraffin capsule shell. This filling process enhanced the compactness of the capsule structure, reducing the likelihood of core material loss and improving the encapsulation and compactness on epoxy resin.

Microcapsules are carrier systems composed of an external shell and an internal core material. The sealing performance of the shell on the core material is an important parameter that affects the stability and release behavior of the core material in microcapsules. Researchers have focused on optimizing the sealing performance of microcapsule shells through various approaches. Firstly, the properties of the shell material, such as polymer type, molecular weight, and crosslinking degree, have been modified to enhance the sealing performance of the shell. Secondly, it has been found that the sealing performance of microcapsules can be improved by adjusting the structural parameters of the shell, including thickness, porosity, and surface morphology. Additionally, nanotechnology has been utilized to prevent core material leakage by encapsulating the active substances within nanoparticles and then enclosing these nanoparticles within the microcapsules. In summary, research on the sealing performance of microcapsule shells on the core material is continuously advancing. By focusing on material selection, structural design, and innovative encapsulation techniques, researchers aim to enhance the sealing performance of microcapsules to meet the diverse application requirements in various fields. However, further studies are needed to address issues such as shell stability, size uniformity, and long-term storage stability to achieve superior sealing performance.

### 3.3. Particle Size Distributions

**Figure 6:**
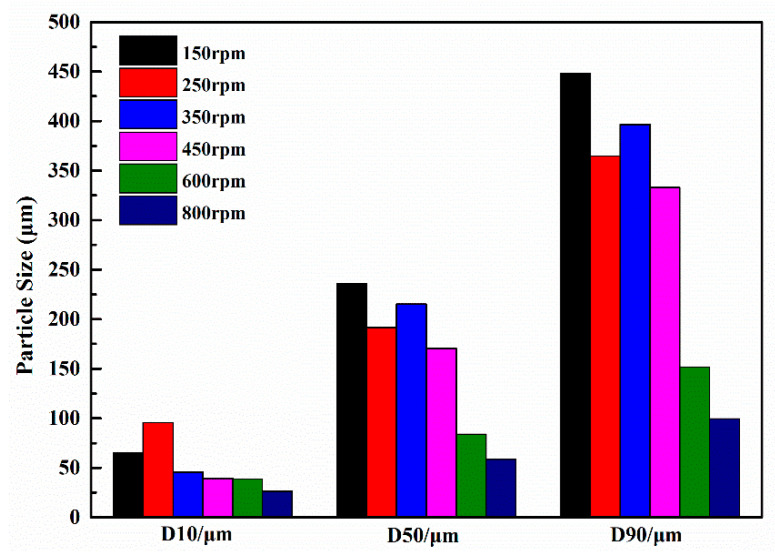
Particle size distribution of various microcapsules.

In a study conducted by Xu et al. [54], urea-formaldehyde coated epoxy resin microcapsules were prepared using an in situ polymerization method. The researchers examined the influence of different rotation speeds on the particle size of the microcapsules during the synthesis process. The particle size of the microcapsules exhibited a range of 0 to 650 μm within the rotation speed range of 150 to 800 rpm, as illustrated in Figure 6. Subsequent analysis demonstrated a linear correlation between the medium particle size of the microcapsules and the rotation speed. It was observed that microcapsules synthesized at a rotation speed of 450 rpm exhibited a particle size distribution that aligned well with the pore distribution of cemented coral sand mortar. Upon adding microcapsules with a mass ratio of 3% cement, the compressive strength of the mortar increased by approximately 85%. This indicates the effectiveness of incorporating microcapsules in enhancing the compressive strength of the mortar.

**Figure 7:**
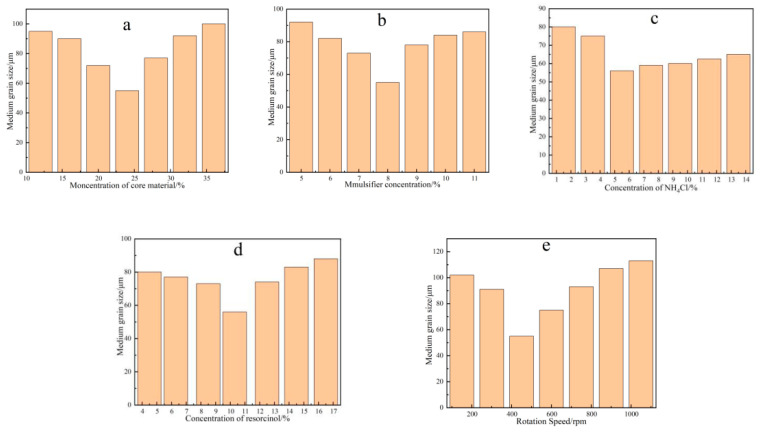
Average particle sizes of microcapsules with different amount of (**a**) epoxy resin; (**b**) SDBS; (**c**) NH4Cl; (**d**) resorcinol; and (**e**) the stirring speed.

Yao et al. [55] utilized the in situ polymerization technique to produce self-healing microcapsules by employing urea-formaldehyde resin as the shell material and E-5l epoxy resin as the core material. The study aimed to investigate the influence of various factors, including the amount of epoxy resin, sodium dodecyl benzene sulfonate (SDBS), ammonium chloride, resorcinol, and stirring rate, on the particle size of the microcapsules. As shown in Figure 7, the optimal conditions for achieving an average particle size of 55.7 μm were determined as follows: a 24% ratio of epoxy resin to urea, 8% SDBS content, 5.6% ammonium chloride content, 10.4% resorcinol content, and a stirring speed of 450 rpm.

Zotiadis et al. [56] successfully prepared microcapsules of polyurea-formaldehyde shells coated with epoxy resin using in situ polymerization method, and the particle size of the microcapsules ranged from 37 to 66 μm. It was shown that the mass ratio of the core/shell material has a significant effect on the particle size of the microcapsules. This is because it determines the core droplet size under constant emulsification conditions, as well as the degree of cross-linking and encapsulation on the shell with a constant number of monomers. The optimal mass ratio of core to shell material is 2:1, when the particle size of microcapsules is 36.8 μm.

**Figure 8:**
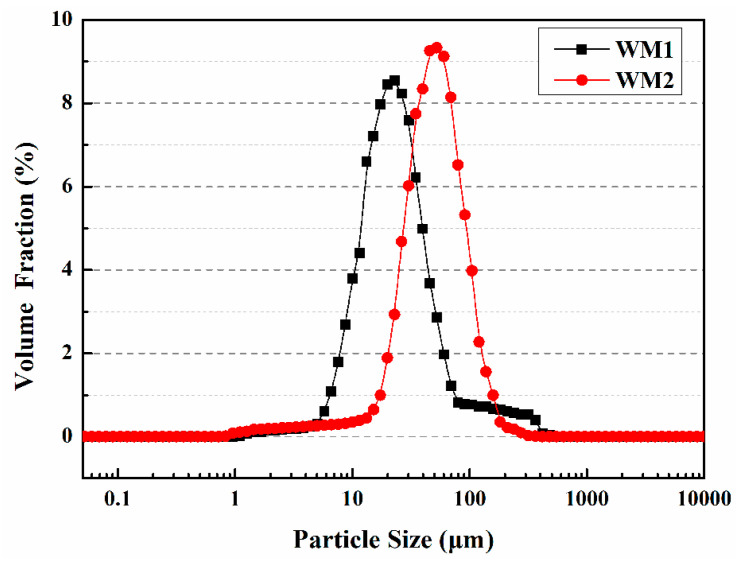
Particle size distributions of microcapsules [57].

Du et al. [57] prepared two types of microcapsules (WM1, WM2) using ceresine wax and nano-CaCO_3_/ceresine wax coated epoxy resin, respectively. The size distribution of WM1 particles ranges from 1 μm to 80 μm, while WM2 particles range from 20 μm to 120 μm, with the majority falling within the 40 μm to 60 μm range (Figure 8). The microcapsules’ D10, D50, and D90 values are provided in Table 1, with average particle sizes of 23 μm for WM1 and 52 μm for WM2. The decreased viscosity of ceresin wax enhances its dispersibility during the mixing process, leading to a reduction in the particle size of WM1. This reduction can be attributed to the application of shear force resulting from the addition of coolant. However, the addition of nano-CaCO_3_ increases the shell mixture’s viscosity, degrading its rheology and deformability, and hindering dispersion during microcapsule preparation. This results in an increase in microcapsule particle size at a constant stirring speed.

The study of particle size distribution of microcapsules is an important research direction as it greatly affects the properties and applications of microcapsules. Currently, laser particle size analyzers are commonly used to determine the particle size distribution of microcapsules. The main factors influencing the particle size distribution of microcapsules include the preparation method, material properties, and reaction conditions. Different preparation methods and reaction conditions can lead to different particle size distributions of microcapsules, and selecting appropriate preparation methods and reaction conditions can achieve the desired particle size distribution of microcapsules. The particle size range of microcapsules is generally believed to below 1000 μm. As microcapsule technology continues to develop, research on particle size distribution is becoming more and more in-depth. Future research directions may include the relationship between particle size distribution and microcapsule properties, improvement of preparation methods, and research on new microcapsule materials.

### 3.4. Thermal Performance

In their study, Zhang et al. [58] successfully utilized an in-situ polymerization method to encapsulate paraffin within modified melamine-formaldehyde (MF) microcapsules, employing hydrophobic silicon carbide (H-SiC) as the shell material. Analysis of the FTIR spectrum confirmed the presence of uniformly distributed embedded H-SiC particles on the surface of the modified microcapsules. A comparative analysis with conventional MF microcapsules revealed a significant 55.82% increase in thermal conductivity for the H-SiC modified MF microcapsules, accompanied by a 10% reduction in melting permeability. Furthermore, the H-SiC modified MF microcapsules exhibited a high melting enthalpy of 93.21 J/g, indicating exceptional thermal storage performance and remarkable thermal stability.

**Figure 9:**
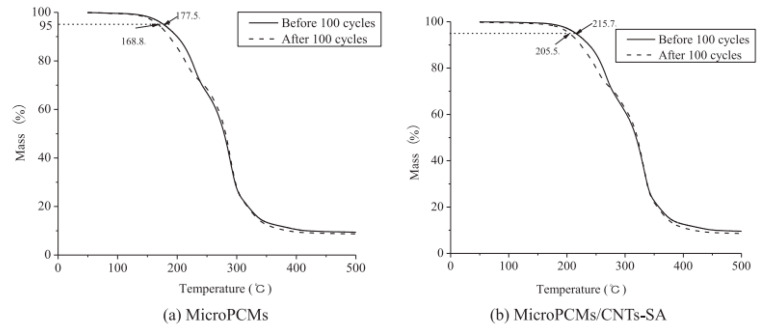
TG curves of (**a**) MicroPCMs; (**b**) MicroPCMs/CNTs-SA before and after 100 cycles [59], Copyright, 2014, Elsevier, License Number 5550141158770.

Li et al. [59] successfully prepared phase change microcapsules via in situ polymerization using urea-formaldehyde resin (UFR) as the shell material, styrene-maleic anhydride copolymer (SMA) as the emulsifier, and carbon nanotubes (CNTs) as the thermal conductivity enhancer. They enhanced the thermal conductivity of the microcapsules by grafting stearic acid onto carbon nanotubes (CNTs-SA) and synthesized phase change microcapsules (MicroPCMs). The phase transition temperature and latent heat of MicroPCMs/CNTs-SA were 26.2 °C and 47.7 J/g, respectively. Compared to MicroPCMs/CNTs, MicroPCMs/CNTs-SA exhibited higher thermal conductivity and mechanical strength. The addition of 4% CNTs increased the thermal conductivity of MicroPCMs/CNTs-SA by 79.2% compared to MicroPCMs with 4% CNTs. Furthermore, the inclusion of CNTs improved the thermal stability of MicroPCMs. MicroPCMs/CNTs-SA demonstrated an initial decomposition temperature that was 38 °C higher than that of MicroPCMs alone. Even after undergoing 100 heating and cooling cycles, MicroPCMs/CNTs-SA displayed excellent durability and thermal stability. However, there was a slight decrease of 4.7% in the initial decomposition temperature of MicroPCMs/CNTs-SA (Figure 9).

**Figure 10:**
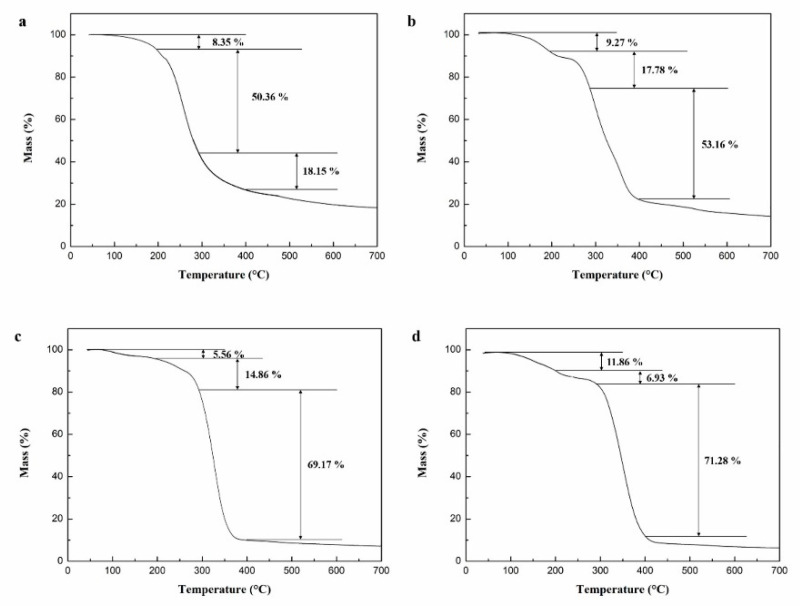
TG curves of microcapsules prepared with different shell/core weight ratios. (**a**) 1.2:1, (**b**) 1:1, (**c**) 1:1.2, (**d**) 1:1.4 [60].

In our previous experiments, microcapsules were prepared by coating N, N-dimethylformamide diluted epoxy resin with microcrystalline wax [60]. To investigate the impact of core/shell ratio on the thermal properties of the microcapsules, experiments were conducted at preparation temperatures of 105 °C and stirring speeds of 900 rpm. Figure 10 illustrates that the mass loss of the microcapsules occurred primarily within three temperature ranges: 100–198 °C, 198–280 °C, and 280–396 °C. The initial two temperature ranges witnessed mass loss due to the melting and decomposition of the microcrystalline wax. However, the protective effect of the microcapsule shell on the core material weakened as a result of shell material damage. The mass loss observed within the temperature range of 280–396 °C resulted from the evaporation of N, N-dimethylformamide and the thermal decomposition of the epoxy resin.

Figure 10 demonstrates the impact of the shell–core weight ratio on the mass loss of the microcapsules across different temperature ranges. In Figure 10a, when the shell–core weight ratio was 1.2:1, the microcapsules exhibited a mass loss reduction of 8.35%, 50.36%, and 18.15% in the temperature ranges of 100–198 °C, 198–280 °C, and 280–396 °C, respectively. Conversely, in Figure 10b, when the shell–core weight ratio decreased to 1:1, the microcapsules demonstrated a mass loss reduction of 9.27%, 17.78%, and 53.16% in the temperature ranges of 100–198 °C, 198–280 °C, and 280–396 °C, respectively. Subsequently, in Figure 10c,d, as the shell–core weight ratio further decreased to 1:1.2 and 1:1.4, the mass loss of the microcapsules increased to 69.17% and 71.28%, respectively, specifically within the temperature range of 280–396 °C. These findings indicate that a decrease in the shell–core weight ratio led to a significant increase in mass loss within the temperature range of 280–396 °C, suggesting a higher encapsulation of epoxy resin within the microcrystalline wax. The optimal weight ratio was determined to be 1:1.2 for microcrystalline wax to epoxy resin.

Research on the thermal performance of microcapsules refers to the performance of microcapsule materials under thermal effects, including thermal stability, thermal conductivity, heat capacity, and phase change energy storage. Currently, research on the thermal performance of microcapsules has made significant progress. The influence of preparation methods and material selection of microcapsule materials on their thermal performance has been studied. The study shows that the thermal stability and thermal conductivity of the microcapsule shell material have a significant impact on the thermal performance of the microcapsules. Additionally, material selection and preparation methods can be used to regulate the thermal performance of microcapsules. In summary, the study of the thermal performance of microcapsules has important theoretical significance and practical application value. With further research, it is expected that more new discoveries and applications will emerge in the field of microcapsule thermal performance.

### 3.5. Micromechanical Properties

**Figure 11:**
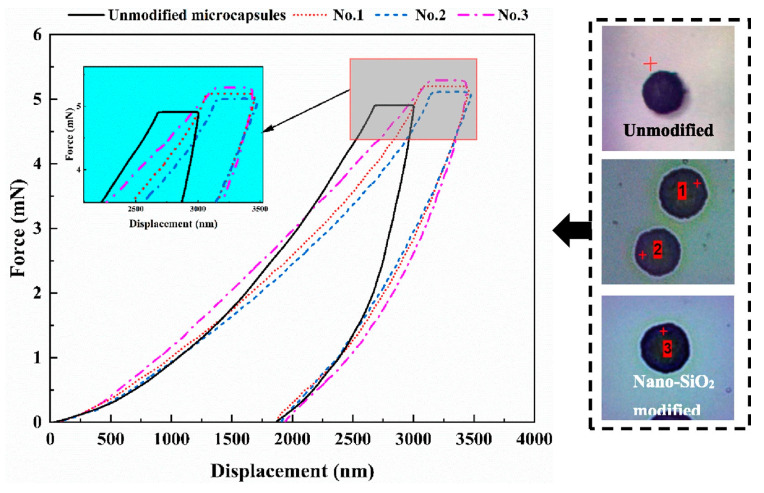
The curves of force–displacement for microcapsules [61], Copyright, 2023, Elsevier, License Number 5550150093739.

Ji et al. [61] prepared microcapsules using in situ polymerization with nano-SiO_2_ modified melamine urea formaldehyde (MUF) as the shell material to encapsulate low-quality vegetable. Nanoscale indentation tests were performed on the unmodified microcapsules and three groups of nano-SiO_2_ modified microcapsules. The Oliver–Pharr analysis method was used to calculate the Young’s modulus and hardness of the microcapsules. The obtained results revealed that the microcapsules exhibited a Young’s modulus ranging from 2.58 to 2.80 GPa, with an average value of 2.63 GPa. This finding suggests that the incorporation of nano-SiO_2_ effectively enhanced the mechanical strength of the microcapsules. Furthermore, the force–displacement curves of the nano-SiO_2_ modified microcapsules exhibited similar changes, indicating that the prepared microcapsules possessed comparable mechanical properties and exhibited good resistance to deformation, as depicted in Figure 11.

Ahangaran et al. [62] conducted nanoindentation tests to investigate the elastic modulus and hardness of PMMA microcapsules loaded with epoxy prepolymer (EC 157) and pentaerythritol tetrakis (3-mercaptopropionate) (PETMP). The elastic modulus of the PMMA shell ranged from 2.4 ± 0.7 to 3.5 ± 0.5 GPa, while the shell hardness varied from 0.064 ± 0.021 to 0.22 ± 0.055 GPa. Comparatively, PMMA microcapsules containing epoxy resin exhibited a higher elastic modulus than those containing mercaptan. The presence of mercaptan acted as a plasticizer, affecting the mechanical strength of the PMMA shell. The study revealed that the molecular weight of PMMA had the most significant impact on the mechanical properties of the healing agent/PMMA microcapsules. Additionally, the average size of the microcapsules, ranging from 5–45 μm, had a relatively minor influence on the mechanical properties of the microcapsule shell.

**Figure 12:**
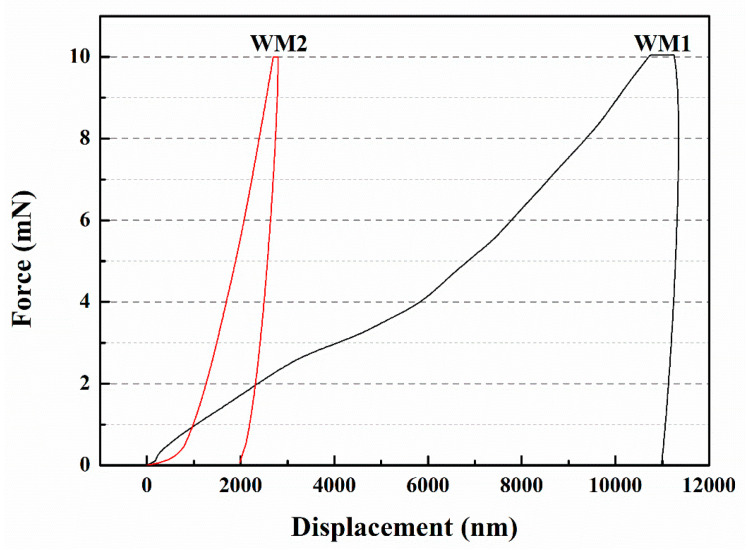
Force–displacement curves of microcapsules [57].

Du et al. [57] fabricated two types of microcapsules, namely WM1 and WM2, utilizing ceresine wax and nano-CaCO_3_/ceresine wax-coated epoxy resin. Nanoindentation testing was conducted to assess the elastic modulus and hardness of the microcapsules, as presented in Table 2. WM1 exhibited an elastic modulus of 0.55 GPa and a hardness value of 4.89 MPa, while WM2 demonstrated higher values with an elastic modulus of 2.02 GPa and a hardness of 72.54 MPa. These findings indicate that WM2 possessed superior mechanical properties compared to WM1. The shell materials play a crucial role in determining the mechanical properties of the microcapsules. Incorporating nano-CaCO_3_ into ceresine wax establishes physical crosslinking points, with the ceresine wax chains enveloping the nano-CaCO_3_ particles. Under stress, the nano-CaCO_3_ facilitates stress transfer among molecular chains, dispersing the load. This “cross-linking-like” effect of nano-CaCO_3_ enables other molecular chains to withstand stress, reducing the likelihood of rapid failure and providing reinforcement. The force–displacement curve of the microcapsules, depicted in Figure 12, illustrates that WM2 exhibited a displacement of 1980 nm, whereas WM1 had a larger displacement of 10,998 nm. The ability of microcapsules to resist plastic deformation induced by pressure depends on their elastic modulus and hardness. Materials with high elastic modulus and hardness experience smaller elastic deformation when subjected to external forces. The addition of nano-CaCO_3_ significantly enhanced the elastic modulus and hardness of the microcapsule shell, thereby reducing the displacement of the microcapsules.

The mechanical properties of microcapsules are an important research focus, encompassing aspects such as mechanical strength, fracture behavior, and deformability. The mechanical strength of microcapsules refers to their resistance to tensile, compressive, or shear forces under external stress. Researchers control factors such as the capsule’s shell material, shell thickness, and fabrication processes to adjust its mechanical strength and meet specific application requirements. The study of fracture behavior in microcapsules examines their fracture modes and fracture toughness under external stress. Through experimental testing and numerical simulations, researchers analyze the fracture behavior of microcapsules to understand their fracture mechanisms and improve fracture performance. The deformability of microcapsules refers to their ability to undergo reversible deformation or exhibit shape memory effects under external stress. Researchers design microcapsule structures with controllable deformability, including reversible deformation or shape memory effects, for applications in programmable materials and micro/nanomechanical systems. In summary, the research on the mechanical properties of microcapsules aims to explore microcapsule materials with excellent mechanical properties and provide a foundation for their engineering applications. Currently, researchers are continuously improving fabrication methods and characterization techniques to achieve precise control and optimization of the mechanical properties of microcapsules.

## 4. The Effect of Microcapsule Addition on the Initial Properties of the Cement-Based Material

### 4.1. Mechanical Properties

**Figure 13:**
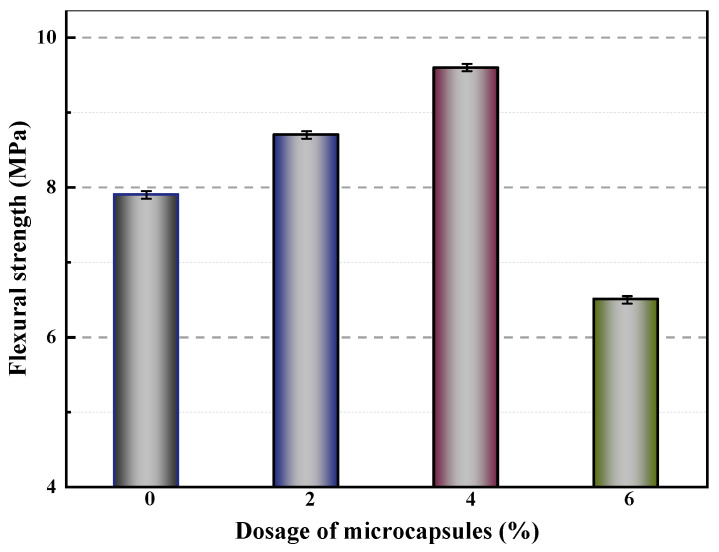
Flexural strength of mortars containing microcapsules [60].

Microcrystalline wax was utilized to coat epoxy resin in the preparation of microcapsules [60]. The flexural strength of mortars containing varying amounts of microcapsules was evaluated, as shown in Figure 13. The control mortar (M-0) had a flexural strength of 7.9 MPa, while mortars with 2% (M-1) and 4% (M-2) cement weight microcapsules exhibited increased flexural strengths of 8.7 MPa and 9.6 MPa, respectively. However, the flexural strength of the mortar exhibited a decline of 17.7% when the microcapsule content was increased to 6% (M-3) compared to the control sample (M-0). These findings indicated that the mechanical properties of the mortar were initially enhanced but then diminished as the microcapsule content increased. This phenomenon can be attributed to the significant disparity in Young’s modulus between the microcapsules and the cement matrix, resulting in inadequate bonding at the interface and the formation of voids within the matrix material. As the microcapsule content increased, the number of pores in the matrix material also increased, resulting in reduced stiffness and decreased flexural strength. However, the addition of a small amount of microcapsules improved the fluidity and compactness of the mortar, resulting in a more compact mortar structure and improved flexural strength.

**Figure 14:**
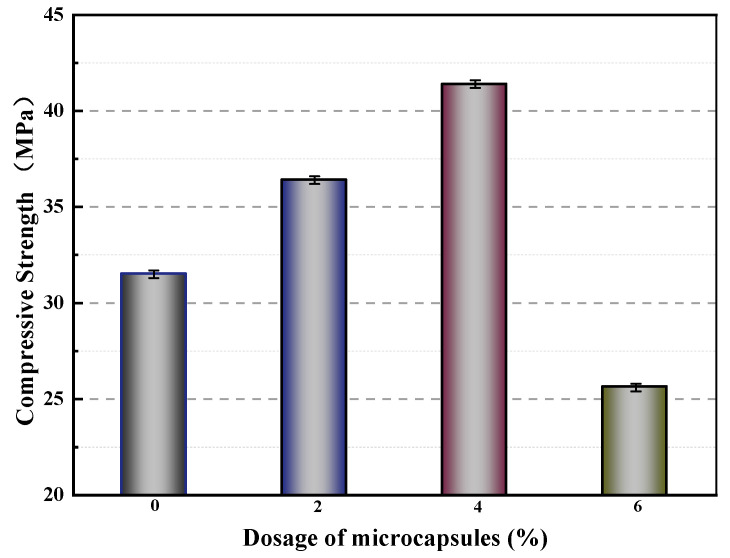
Compressive strength of mortars containing microcapsules [60].

Figure 14 displays the compressive strengths of mortars containing varying amounts of microcapsules after 28 days of standard curing. The compressive strengths of M-0, M-1, M-2, and M-3 were 31.5 MPa, 36.4 MPa, 41.4 MPa, and 25.6 MPa, respectively. Compared to M-0, M-1 and M-2 exhibited increased compressive strengths of 15.6% and 31.4%, respectively. This can be attributed to the presence of voids in the mortar, which can be filled by an appropriate amount of microcapsules, resulting in an improvement in the compressive strength. In contrast, the compressive strength of M-3 decreased by 5.7% compared to that of M-0. This decrease may be due to the significant difference in modulus between the microcapsules and the cement matrix. Excessive amounts of microcapsules in the mortar can result in gaps in the bonding surface between the microcapsules and the matrix material. These gaps reduce the compactness of the mortar during the molding process and affect the particle gradation of the cementitious material, ultimately leading to a decrease in compressive strength.

Microcapsules with polyethylene wax shell (SWMs) and Fe_3_O_4_ nanoparticles/polyethylene wax shell (DWMs) were prepared using the melt dispersion condensation method, with epoxy resin as the healing agent [53]. Table 3 illustrates the flexural strengths and compressive strengths of SJ-0, SJ-1, and SJ-2. The flexural strengths measured for SJ-0, SJ-1, and SJ-2 were 8.2 MPa, 7.9 MPa, and 7.5 MPa, respectively. Compared to SJ-0, SJ-1 exhibited a 3.7% reduction in flexural strength, while SJ-2 showed an 8.5% reduction. The compressive strengths for SJ-0, SJ-1, and SJ-2 were determined as 33.4 MPa, 32.6 MPa, and 30.9 MPa, respectively. Similarly, SJ-1 and SJ-2 demonstrated reductions of 2.3% and 7.5% in compressive strength, respectively, compared to SJ-0. These changes can be attributed to the significant difference in Young’s modulus between the cement matrix and the microcapsule shell. The incorporation of microcapsules into cement mortar leads to the formation of gaps at the interfaces between the microcapsules and the cement matrix, resulting in increased porosity within the matrix and a decrease in stiffness, flexural strength, and compressive strength of the mortar. Furthermore, the addition of microcapsules affects the compactness of the mortar during the molding process and disrupts the particle gradation, leading to decreased flexural strength and compressive strength.

In reference [57], two types of microcapsules, named WM1 and WM2, were developed using ceresine wax and nano-CaCO_3_/ceresine wax-coated epoxy resin, respectively. The compressive strength of mortar is presented in Table 4. CCM0, CCM1, and CCM2 had compressive strengths of 31.2 MPa, 40.6 MPa, and 35.5 MPa, respectively. The findings indicated that the appropriate addition of microcapsules significantly enhanced the compressive strength of the mortar. This enhancement was evident from the increase in compressive strengths of CCM1 and CCM2 by 30.1% and 13.8%, respectively, compared to CCM0. The improvement can be attributed to the ability of microcapsules to fill the internal pores of the mortar, thereby enhancing its compressive strength. It is worth noting that CCM1 exhibited higher compressive strength than CCM2. This can be attributed to the smaller particle size of WM1, which enables better filling of the internal pores of the mortar and reduces the presence of microdefects.

In general, the addition of microcapsules can have a significant impact on the mechanical properties of cement-based materials. At an appropriate dosage, microcapsules can fill the internal pores of the cement-based material, improving its flexural and compressive strength. However, excessive addition of microcapsules can lead to gaps in the bonding surface between the capsules and matrix material, reducing the compactness of the cement-based material and affecting particle gradation during the molding process, thereby negatively impacting the mechanical properties of the cement-based material. Additionally, the properties of the microcapsules, such as particle size, can also affect the mechanical properties of the cement-based material by influencing their ability to fill internal pores and reduce micro-defects. Therefore, careful selection and control of the dosage of microcapsules is crucial in achieving optimal improvements in mechanical properties of cement-based materials.

### 4.2. Pore Size Distribution

Pores smaller than 0.1 μm in size are generally regarded as harmless, or at least not significantly harmful, while those larger than 0.1 μm can have a negative impact on both the mechanical properties and permeability of cement-based materials. Consequently, this section will focus on pores larger than 0.1 μm in size, which are deemed harmful.

**Figure 15:**
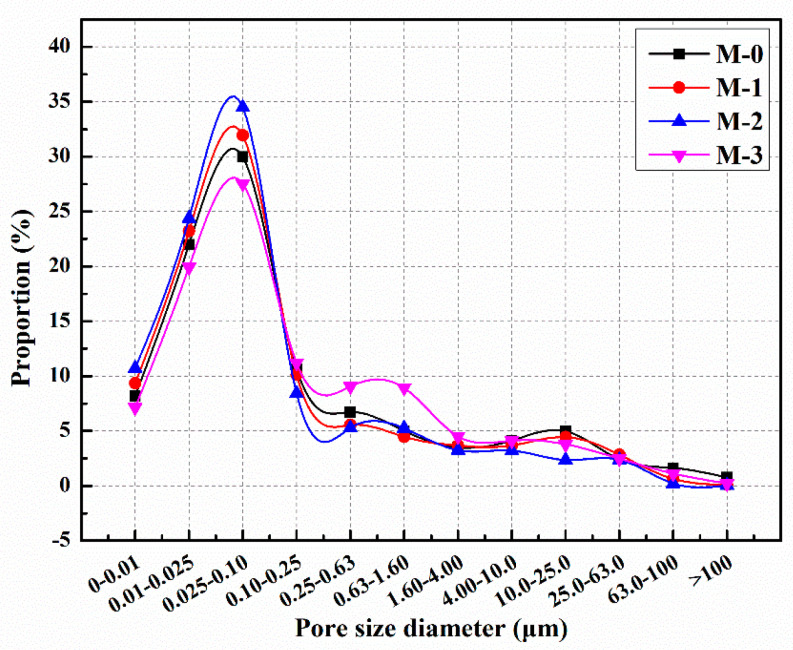
Pore size distribution of cement mortars with microcapsules [60].

The distribution of pore sizes in mortars with different microcapsule contents is illustrated in Figure 15 [60]. It can be observed that the proportion of harmful pores in M-0, M-1, M-2, and M-3 was 39.8%, 35.5%, 30.4%, and 45.4%, respectively. In comparison to M-0, the proportion of harmful pores in M-1 and M-2 decreased by 10.8% and 23.6%, respectively. These findings indicate that the addition of microcapsules enhanced the compactness of the cement mortar and reduced the proportion of harmful pores. Cement mortar consists of various materials with different particle sizes, and the incorporation of an appropriate amount of microcapsules can optimize the particle gradation and internal structure of the mortar, leading to increased compactness. However, the proportion of harmful pores in M-3 increased by 14.7% compared to M-0. This suggests that excessive microcapsule content can result in the formation of gaps at the bonding interface between the microcapsules and the cement matrix, thereby reducing the compactness of the mortar and increasing the proportion of harmful pores.

**Figure 16:**
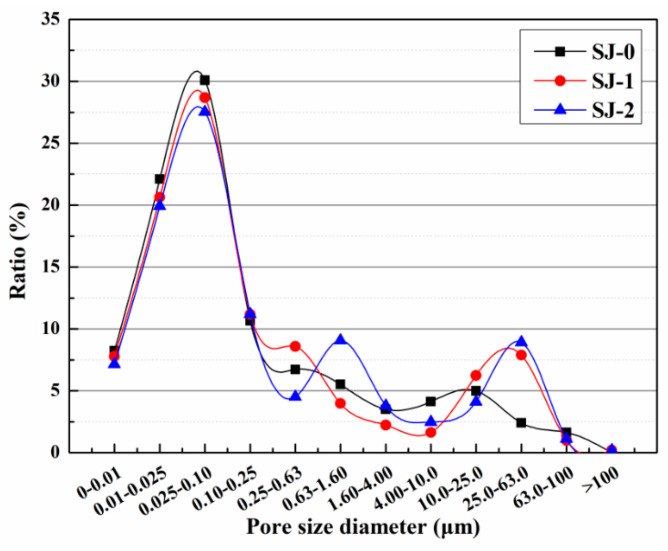
Pore size distribution of SJ-0, SJ-1, SJ-2 [53].

The harmful pore ratios of SJ-0, SJ-1, and SJ-2 are depicted in Figure 16. The harmful pore ratios were found to be 39.57%, 42.92%, and 45.41% for SJ-0, SJ-1, and SJ-2, respectively. In comparison to SJ-0, SJ-1 exhibited an increase in the harmful pore ratio by 3.35%, while SJ-2 showed an increase of 5.84%. This can be attributed to the larger size of SWMs and DWMs, which leads to an enlargement of the internal pore size within the mortar. Consequently, the compactness of the mortar is reduced, resulting in a higher harmful pore ratio. Harmful pores refer to the voids within mortar that have a negative impact on its strength and durability, potentially leading to issues such as chloride ion penetration, chemical erosion, and freeze–thaw cycles. Consequently, an increase in the proportion of harmful pores can result in a decrease in the strength and durability of the mortar. Therefore, when designing and preparing mortar, it is crucial to consider the selection of particle sizes in order to strike a balance between the compactness and performance requirements of the mortar. Further research could explore alternative approaches to improve the compactness and resistance to permeability of mortar, addressing the issue of harmful pores.

**Figure 17:**
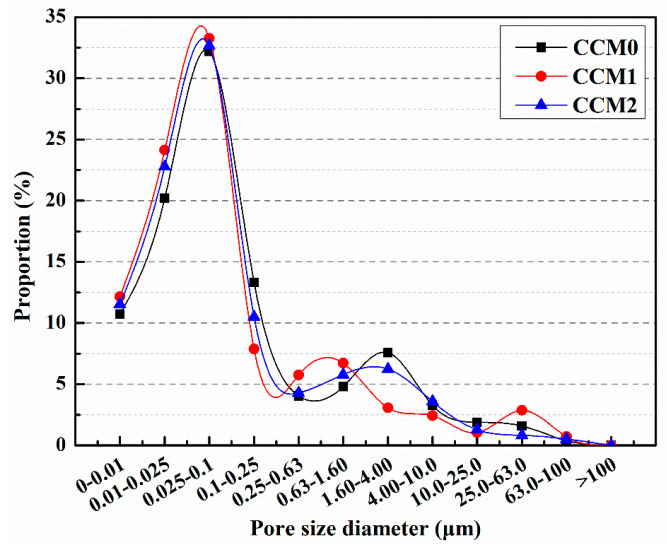
Pore size distribution of mortars.

The pore size distribution of mortar is depicted in Figure 17 [57]. The proportion of harmful pores for CCM0, CCM1, and CCM2 was measured as 36.89%, 30.44%, and 33.04%, respectively. Incorporating microcapsules into the mortar led to a reduction in the proportion of harmful pores, with CCM1 experiencing a decrease of 17.5% and CCM2 experiencing a decrease of 10.4% compared to CCM0. This indicates that the addition of microcapsules improved the compactness of the mortar. Mortar consists of various particles of cement, sand, and water, resulting in the presence of pores in the final product. By introducing microcapsules during the preparation of the mortar, some of these pores and microdefects can be filled, thereby enhancing the internal structure and compactness of the mortar and reducing the proportion of harmful pores. Mortar is composed of various particles such as cement, sand, and water, resulting in the presence of pores in the final product. Introducing microcapsules during the preparation of mortar can fill some of these pores and microdefects, thereby enhancing the internal structure and compactness of the mortar and reducing the proportion of harmful pores. Future research could further explore the optimization design of microcapsules and suitable additives to achieve better improvements in mortar performance. In addition, more in-depth research and experiments are needed to investigate the selection and use of microcapsules for different types of mortar and specific application scenarios. This will contribute to further optimizing the structure and performance of mortar and promote the development and innovation in the field of building materials.

The addition of microcapsules has a significant impact on the pore size distribution of mortar. The pore size distribution of mortar plays a crucial role in its performance and durability, as different pore sizes can affect permeability, strength, and resistance to freeze–thaw cycles. By introducing microcapsules, a portion of the pores and microdefects in the mortar can be filled. These microcapsules have a sealed outer shell and contain various functional materials such as enhancers, fillers, or protectants. During the curing process of the mortar, these microcapsules rupture and release their contents, which can fill the voids in the mortar and interact with cement and other particles. The addition of microcapsules allows for adjustment and improvement of the pore size distribution in mortar. Smaller microcapsules can fill smaller voids, while larger microcapsules can fill larger voids. As a result, the range of pore size distribution narrows, making the pores in the mortar more uniform and compact. Furthermore, the filling of voids can reduce microdefects in the mortar and enhance the overall structural integrity. By adjusting the pore size distribution of the mortar, the addition of microcapsules can reduce the proportion of harmful pores. These harmful pores can lead to the penetration of moisture, gas, and chemicals, thereby reducing the strength and durability of the mortar. By filling and reducing the harmful pores, the introduction of microcapsules improves the compactness and resistance to permeability of the mortar. Therefore, the addition of microcapsules has a positive impact on the pore size distribution of mortar, improving its structure and performance. This opens up new possibilities for the application of mortar in the field of construction and engineering and contributes to further optimization and innovation of building materials.

## 5. The Self-Healing Mechanism and Effect of Microcapsules

### 5.1. Physical Trigger

#### 5.1.1. Mechanical Fracture Trigger

**Figure 18:**
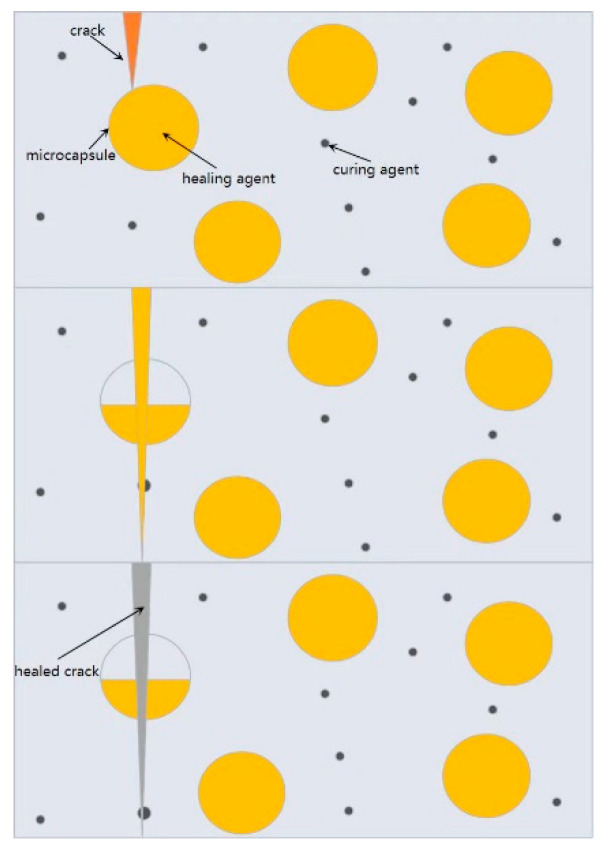
The self-healing processes.

When cracks occur within cement-based materials, the microcapsules’ shells are subjected to stress and rupture. This leads to the release of the encapsulated healing agent contained within the microcapsules. Through thermodynamic or kinetic processes, the healing agent migrates to the crack site and undergoes self-curing or reacts with air, water, or other substances present in the environment, resulting in solidification (Figure 18). Alternatively, when in contact with catalysts infiltrating the cement-based material, the healing agent can trigger further polymerization reactions, thereby healing the cracks. This mechanism describes the self-healing process initiated by mechanical fracture, observed in cement-based materials [63].

#### 5.1.2. Temperature Trigger

Ryu et al. [64] introduced a microfluidic technique that utilizes palm oil as a biocompatible and temperature-responsive shell material for microcapsules. This innovative design enables efficient sealing and controlled release of encapsulated active substances triggered by temperature changes. Unlike conventional paraffin materials such as eicosane, microcapsules with a palm oil shell exhibit excellent sealing properties without the formation of pores or cracks during the freezing process. This provides a highly effective barrier, isolating and safeguarding various complex cargoes, including highly diffusive small molecules, strong acids, cosmetic active substances, and even niacinamide, from the external environment for an extended duration. Moreover, the palm oil shell melts above a specific temperature, enabling precise and controlled release of the encapsulated active substances as required.

#### 5.1.3. Light Trigger

Bartosz et al. [65] introduced a novel approach for reversible and light-responsive microcapsules, offering promising applications in controlled release of drugs, fragrances, and pesticides. They developed microcapsules with polyamide shells containing ortho-substituted azo-benzene, which undergoes trans–cis isomerization upon visible light illumination. The microcapsules were prepared through an oil-water interface polymerization method, and their surface and cross-sectional morphology were examined using scanning electron microscopy. Fragrance release triggered by light irradiation was monitored using gas chromatography-mass spectrometry. By incorporating ortho-substituted azobenzene into the polyamide shell, the researchers successfully modified the shell morphology, resulting in enhanced permeability and release of the encapsulated substance under visible light. The fragrance release mechanism was attributed to the reduction in length of the modified azobenzene upon light exposure. This light-induced compression of the microcapsules offers a unique approach for precisely regulating the release of encapsulated materials.

### 5.2. Chemical Trigger

#### 5.2.1. pH Trigger

In the work by Wang [66], a polyamide microcapsule with dual acid/base-responsive properties and high loading capacity was successfully developed using interfacial polymerization. The preparation process is straightforward, making it suitable for large-scale production. By adjusting the feed ratio of the amine monomer, the permeability of the shell can be controlled, allowing for easy modulation of the release profile. These microcapsules exhibit the ability to retain volatile core materials in a dry or hydrophobic state for an extended period, while enabling pH-dependent release of small molecules over several days. With their versatile pH-responsive behavior, these microcapsules hold promise for various applications, including fragrance release on moist skin surfaces, alkaline laundry processes, and the delivery of pest control agents in agricultural settings.

#### 5.2.2. Ion Trigger

**Figure 19:**
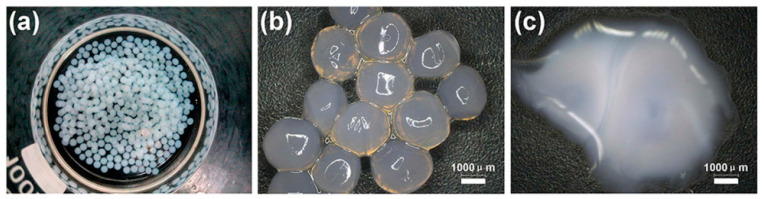
(**a**) Ag-alg capsules (**b**) Optical image of Ag-alg capsules (**c**) The Ag-alginate capsule that disintegrated when exposed to chloride ions [67].

The design of ion-triggered microcapsules aims to address the issue of corrosion of steel reinforcement caused by chloride ions in concrete. Typically, corrosion inhibitors for steel reinforcement are encapsulated within the microcapsules to mitigate the problems of concrete cracking and steel corrosion. The most commonly used materials for the microcapsule shell are polymer ligands and metal ion coordination compounds. The metal ions react with external ions to form precipitates or soluble complexes, thereby altering the porosity of the capsules and releasing the core material. The commonly designed triggering ions include chloride ions, carbonate ions, and sulfate ions. Earlier studies by Xing et al. focused on ion-triggered self-healing microcapsules for concrete [67]. To address chloride ion-induced corrosion, alginate-silver was used as the shell material to achieve chloride ion-triggered processes. Chloride ions can react with silver ions in the shell material, leading to the formation of silver chloride precipitates and subsequent dissolution of the shell material. The morphological changes during the triggering process of the capsules are illustrated in Figure 19.

### 5.3. The Self-Healing Effect of Microcapsules on Cement-Based Materials

In our previous research, we examined the effects of these microcapsules on various aspects of the self-healing effects of mortar. We analyzed the distribution of pore sizes to understand how the presence of microcapsules can restore the pore structure of the mortar. In addition, we evaluated the mechanical properties of the mortar to determine whether the microcapsules can restore them of the damaged mortar. Finally, we examined the self-healing ability of surface cracks in the mortar, with a particular focus on how the microcapsules can promote the self-healing process. It is important to note that the microcapsules were designed to rupture in two different ways: through mechanical fracture triggers and through the application of an external magnetic field for electromagnetic release. These distinct rupture mechanisms allowed us to compare and analyze their respective effects on the self-healing of the mortar.

#### 5.3.1. Pore Size Distribution

**Figure 20:**
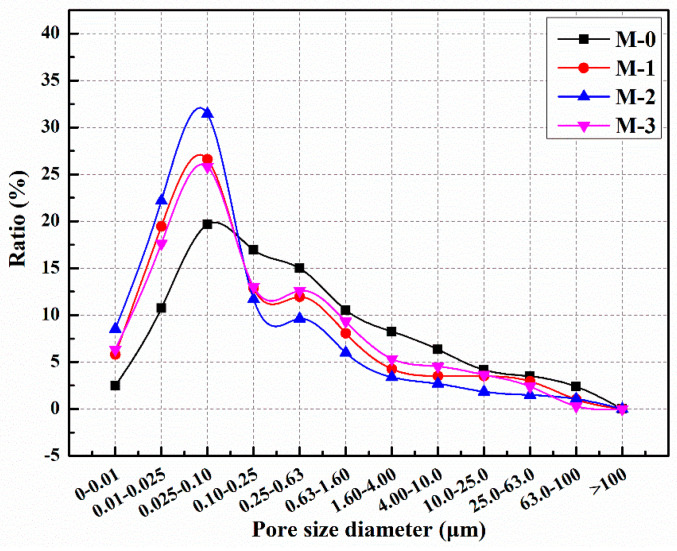
Pore size distribution of cement mortar with microcapsules after self-healing for 14 days.

After the standard 28-day curing period, the compressive strength of the mortar was measured as f_b0_. To create pre-damaged mortar specimens, a load of 60% f_b0_ was applied. These pre-damaged specimens were then kept in a laboratory environment at 20 °C and 50% relative humidity for a self-healing period of 14 days [60]. Following the self-healing process, the pore size distribution of samples with varying microcapsule contents was analyzed, as shown in Figure 20. The proportions of harmful pores in M-0, M-1, M-2, and M-3 were determined to be 67.1%, 48.1%, 37.8%, and 51.2%, respectively. These findings indicate a notable reduction in the proportion of harmful pores in the mortar containing microcapsules after the self-healing period. The decrease in harmful pores can be attributed to the formation of microcracks in the mortar due to preloading. The stress at the crack tips damages the microcapsule shells, resulting in the release of E-51 epoxy resin. The released resin reacts with 2-ethyl-4-methylimidazole present in the mortar, leading to crack filling, pore size refinement, improved mortar compactness, and ultimately a decrease in the proportion of harmful pores.

**Figure 21:**
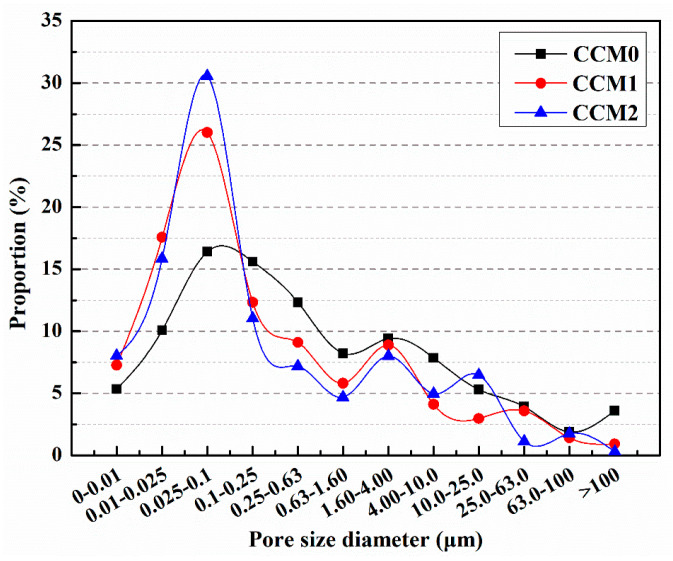
Pore size distribution of mortars after 14 days of self-healing [57].

Following the standard 28-day curing period, six specimens were randomly chosen from each group for mortar compressive strength testing, and the average value was determined as f_a_, representing the compressive strength of the mortar. Subsequently, the mortar specimens were loaded with 60% f_a_ to induce pre-damage. These pre-damaged specimens were then placed in a laboratory environment with a temperature of 25 °C and a relative humidity of 50% for a self-healing period of 14 days. The pore size distribution of the pre-damaged mortar samples after self-healing was analyzed, and the proportions of harmful pores were measured as 68.17%, 49.15%, and 45.54% for CCM0, CCM1, and CCM2, respectively (Figure 21) [57]. Notably, the inclusion of microcapsules in the mortar resulted in a significantly reduced proportion of harmful pores after the self-healing process. The reduction in the proportion of harmful pores can be attributed to the development of internal cracking within the mortar due to pre-loading (microcapsule rupture, self-heal products filling the pores).

**Figure 22:**
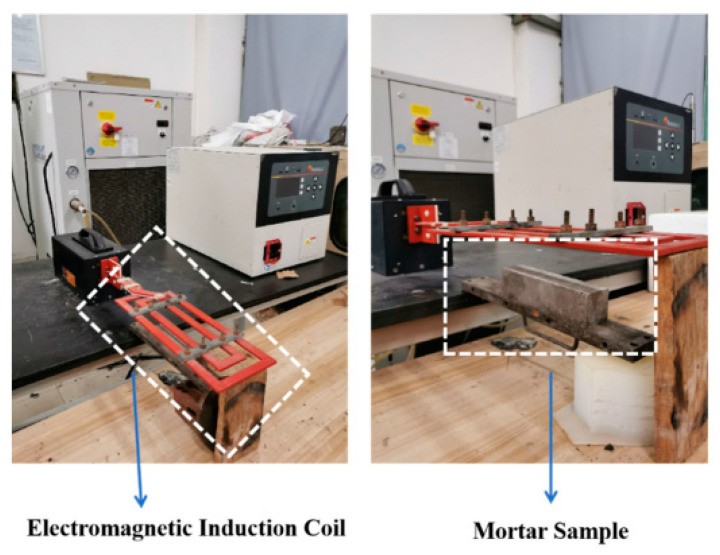
Electromagnetic induction heating system [53].

After the standard 28-day curing period, the compressive strengths of the mortars were measured and represented as f_2_. To induce pre-damage in the mortar samples, a load of 60% f_2_ was applied. Subsequently, SJ-0 and SJ-1 samples were kept at room temperature for a duration of 15 days. In contrast, SJ-2 samples were exposed to an electromagnetic field using an electromagnetic induction heater, as illustrated in Figure 22. The heating process involved subjecting SJ-2 to the electromagnetic field for 45 min with 30-s heating intervals. After the heating process, SJ-2 samples were left at room temperature for an additional 15 days. It is important to note that the electromagnetic field was generated using the electromagnetic induction heater, with the heating coil positioned 0.5 cm away from the upper surface of SJ-2. The electromagnetic induction heater operated at a frequency of 124 kHz, and the output voltage of the equipment was set at 600 V [53].

**Figure 23:**
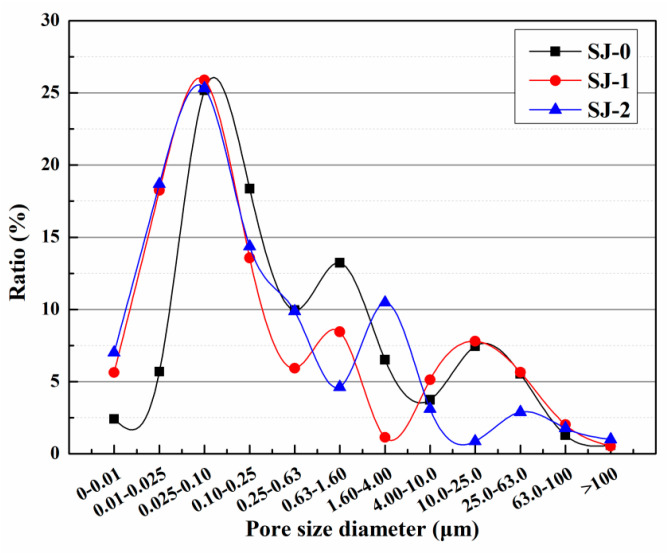
Pore size distribution of mortars after self-healing [53].

The proportions of harmful pores in the pre-damaged SJ-0, SJ-1, and SJ-2 specimens were analyzed after a 15-day self-repairing period, as shown in Figure 23 [53]. The harmful pore ratios were determined to be 66.72%, 50.23%, and 48.97% for SJ-0, SJ-1, and SJ-2, respectively. These results indicate a significant decrease in the harmful pore ratios of SJ-1 and SJ-2, approaching the initial value, after the self-healing process. The reduction can be attributed to the presence of internal cracks within the mortar caused by pre-damage. During crack propagation, the microcapsules are encountered, and the stress at the crack tip can cause their rupture. This leads to the release of epoxy resin, which acts as the healing agent, and reacts with the 2-ethyl-4-methylimidazole, acting as the curing agent. The resulting healing products fill the cracks, reducing the harmful pore ratios. However, it is important to note that the presence of microcapsules within the crack propagation path is not guaranteed, and in such cases, the cracks may not be effectively healed. On the other hand, SJ-2 incorporates microcapsules (DWMs) with Fe_3_O_4_ nanoparticles on the surface. These nanoparticles possess excellent magnetic permeability, generating more heat in the electromagnetic field and minimizing hysteresis loss. The ferromagnetic nature of Fe_3_O_4_ nanoparticles also induces Joule heat due to eddy current loss. When subjected to the applied electromagnetic field, the temperature of the microcapsule shell material increases, causing it to melt and allowing the epoxy resin inside the microcapsules to flow out and react with the 2-ethyl-4-methylimidazole, effectively filling the cracks. This enhanced self-healing ability of the microcapsules results in a reduction in the harmful pore ratios within the mortar.

#### 5.3.2. Compressive Strength Recovery Ratio

**Figure 24:**
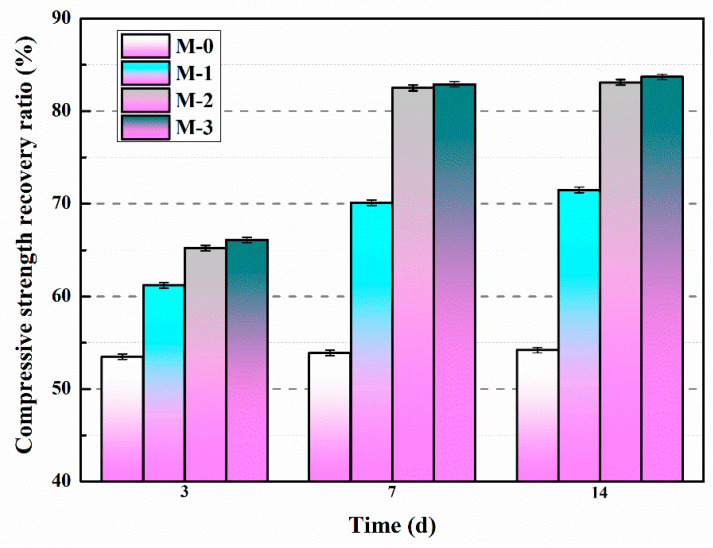
Compressive strength recovery ratios of mortars containing microcapsules [60].

The compressive strength recovery ratios of mortars with different amounts of microcapsules and varying self-healing durations were investigated, as shown in Figure 24 [60]. The study found that the compressive strength recovery ratio of M-0 remained constant regardless of the self-healing time, indicating that the inherent self-healing capability of cementitious materials was insufficient to effectively repair the pre-damaged M-0 within a 14-day period. After a self-healing duration of 14 days, the compressive strength recovery ratios for M-0, M-1, M-2, and M-3 were determined as 54.2%, 71.5%, 83.1%, and 83.7%, respectively. These findings clearly demonstrated that the compressive strength recovery ratios of the mortars increased with higher microcapsule content. However, once the microcapsule content exceeded 4%, the variation in the compressive strength recovery ratio became minimal, suggesting that the microcapsules had reached their limit in effectively addressing the internal microcracks within the mortar.

**Figure 25:**
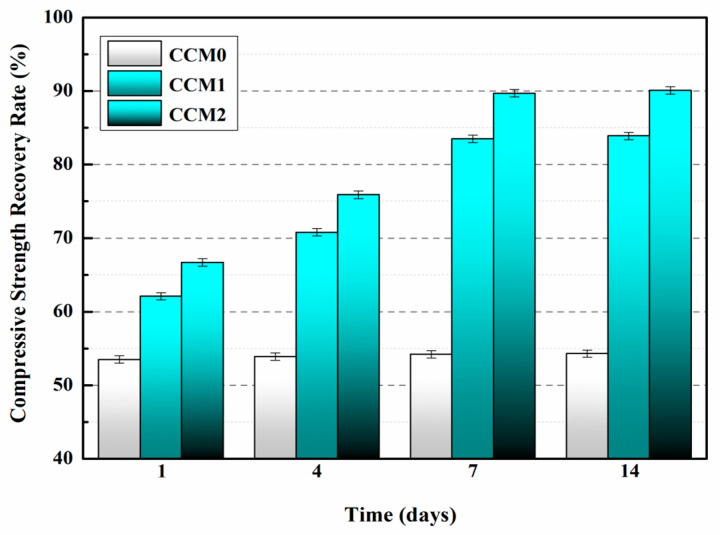
Compressive strength recovery rates of mortars [57].

The variation in the compressive strength recovery rate of the mortar with increasing self-healing time is presented in Figure 25 [57]. It is evident from the results that the compressive strength recovery rate of CCM0 remains constant even with extended self-healing time, indicating the insufficient intrinsic self-healing capability of cement-based materials to repair the pre-damaged CCM0 within a 14-day period. After 14 days of self-healing, the compressive strength recovery rates of CCM0, CCM1, and CCM2 were determined as 54.3%, 83.9%, and 90.1%, respectively. These findings highlight the superior self-healing ability of WM2 for pre-damaged mortar.

**Figure 26:**
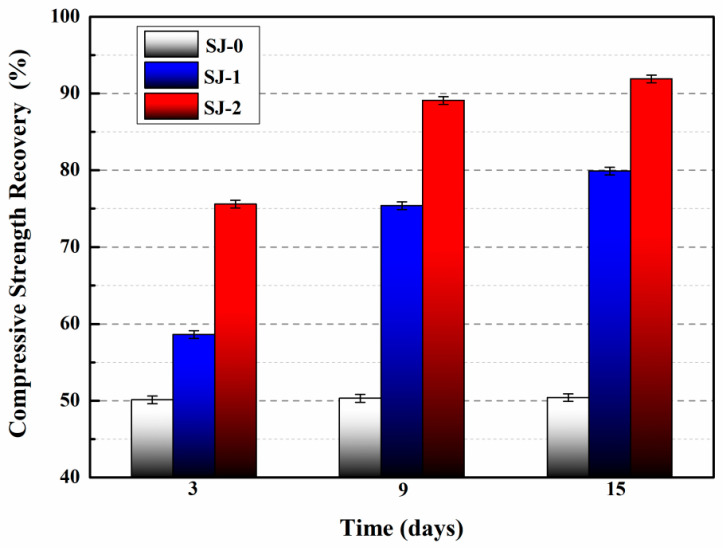
Compressive strength recovery of mortars [53].

The compressive strength recovery of mortars is presented in Figure 26 [53]. For SJ-0, the compressive strength recovery rates at 3, 9, and 15 days were 50.1%, 50.3%, and 50.4%, respectively. These findings suggest that the intrinsic self-healing ability of the cementitious material is insufficient to effectively repair the pre-damaged SJ-0 within the given time frame. In contrast, SJ-1 and SJ-2 exhibited improved compressive strength recovery rates of 79.9% and 91.9%, respectively, after a self-healing period of 15 days. These results indicate significant enhancements compared to SJ-0. Additionally, SJ-2 demonstrated a higher recovery of compressive strength compared to SJ-1, suggesting faster self-healing and improved mechanical properties recovery.

#### 5.3.3. Surface Cracks Self-Healing

**Figure 27:**
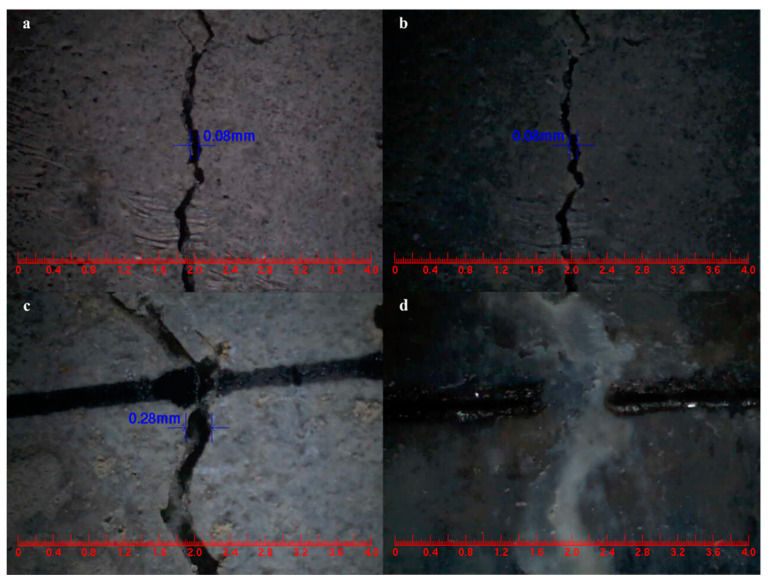
Self-healing of surface cracks in mortar. (**a**) Control mortar, (**b**) control mortar after 3 days self-healing, (**c**) mortar containing microcapsules, (**d**) mortar containing microcapsules after 3 days self-healing [60].

The main goal of this study was to provide direct evidence for the self-healing ability of cement-based materials that incorporate microcapsules, aiming to achieve effective healing properties in the presence of cracks. The mortar composition included a microcapsule content of 4% of the cement weight. Comparing Figure 27a,b, the control mortar displayed an initial surface crack width of 0.08 mm, which remained unchanged after 3 days of self-healing [60]. In contrast, Figure 27c,d illustrate the microcapsule-containing mortar, which exhibited a surface crack with an initial width of 0.28 mm and underwent self-healing within 3 days. These findings demonstrate that the mortar incorporating microcapsules demonstrated rapid self-healing of surface cracks with widths below 0.28 mm.

**Figure 28:**
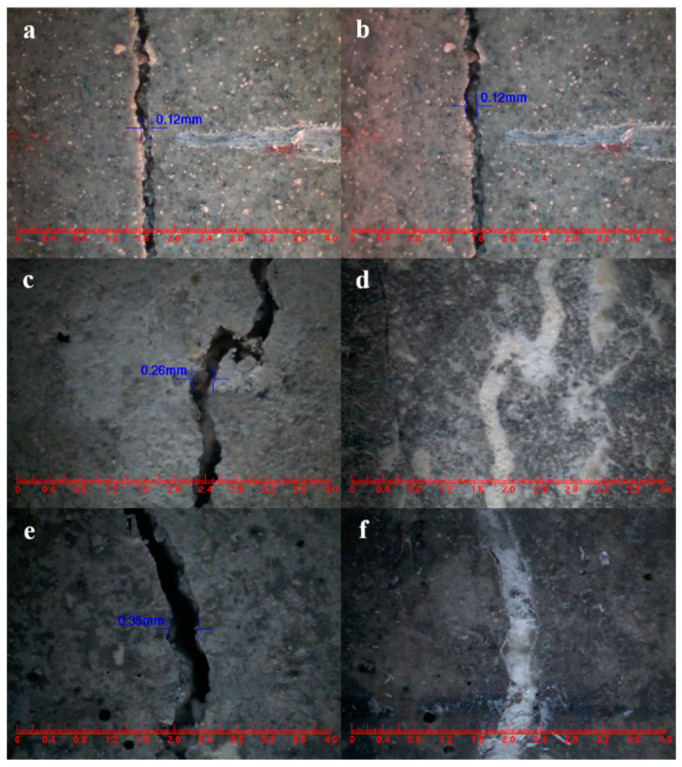
Surface crack self-healing of mortars. (**a**) CCM0 before self-healing, (**b**) CCM0 for 3 days of self-healing, (**c**) CCM1 before self-healing, (**d**) CCM1 for 3 days of self-healing, (**e**) CCM2 before self-healing, (**f**) CCM2 for 3 days of self-healing [57].

The widths of the mortar cracks were measured before and after the self-healing process [57]. Analyzing Figure 28a,b shows that the surface crack in CCM0, initially measuring 0.12 mm in width, remained unchanged after 3 days. This indicates that the intrinsic self-healing ability of the cement-based material was insufficient to repair the surface cracks effectively. In contrast, the surface cracks in CCM1 and CCM2, with initial widths of 0.26 mm and 0.35 mm, respectively, completely self-healed within 3 days, as illustrated in Figure 28d,f. These results demonstrate the effective self-healing capacity of the microcapsules in the mortar. After 3 days, the self-healed width of CCM2 was wider than that of CCM1. This can be attributed to the higher compactness and initial core content of WM2 compared to WM1, resulting in a broader self-healing width for cracks in CCM2. Moreover, WM2 exhibits favorable micromechanical properties, reducing the risk of fracture during mortar mixing and preventing the loss of E-44 epoxy resin. These factors contribute to the successful healing of wider cracks in CCM2.

**Figure 29:**
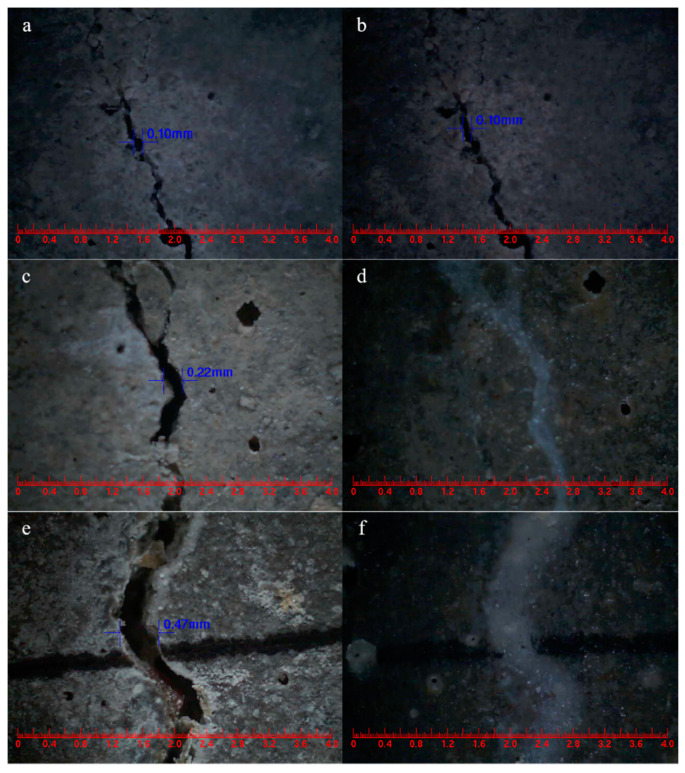
Surface cracks width of mortars. (**a**) SJ-0, (**b**) SJ-0 for 15 days self-healing, (**c**) SJ-1, (**d**) SJ-1 for 15 days self-healing, (**e**) SJ-2, (**f**) SJ-2 for 15 days self-healing [53].

Surface cracks were intentionally introduced on SJ-0, SJ-1, and SJ-2 specimens using the three-point bending method, and their initial widths were measured [53]. After a 15-day self-healing period, the crack widths were measured again, and the results are presented in Figure 29. Analysis of Figure 29a,b indicates that the crack width in SJ-0, which initially measured 0.1 mm, remained unchanged before and after self-healing. This indicates that the cement-based material alone lacks the ability to self-heal its surface cracks. However, the surface crack with an initial width of 0.22 mm in SJ-1 (Figure 29c,d) and 0.47 mm in SJ-2 (Figure 29e,f) completely healed after 15 days. This demonstrates the effective self-healing capability of the microcapsules in the mortar, with SJ-2 exhibiting the ability to heal wider cracks compared to SJ-1.

#### 5.3.4. Frost Resistance and Self-Healing Ability of Concrete (Freeze–Thaw Cycle)

**Figure 30:**
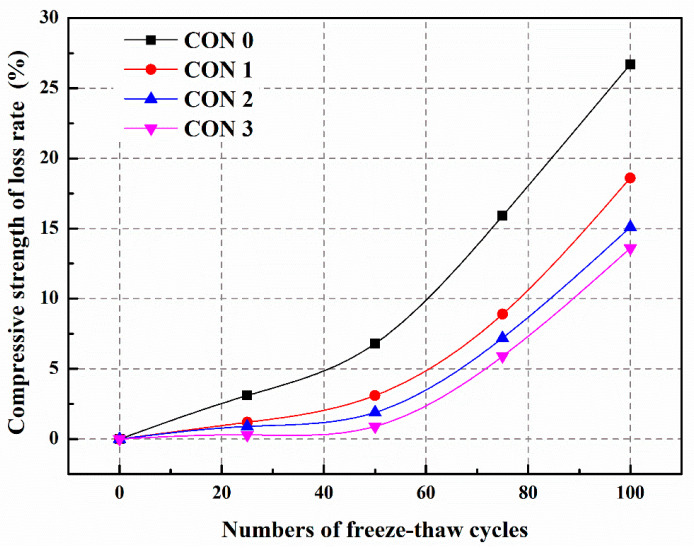
The compressive strength loss rates of concretes during freeze–thaw cycles [39], Copyright, 2021, Elsevier, License Number 5567380935164.

We prepared three different types of microcapsules (Table 5) and incorporated them into C30 concrete (Table 6). The frost resistance and self-healing ability of these concretes were studied through 100 cycles of freeze–thaw testing [39]. From Figure 30, it can be observed that the compressive strength of the concrete significantly decreases after freeze–thaw cycles. This is because microcracks develop within the concrete due to the testing, leading to internal structural damage and ultimately deterioration of the mechanical properties. After 100 freeze–thaw cycles, the compressive strength loss rates for CON0, CON1, CON2, and CON3 are 26.7%, 18.6%, 15.1%, and 13.6%, respectively. Compared to CON0, the compressive strength loss rates of CON1, CON2, and CON3 decrease by 30.3%, 43.4%, and 49.1%, respectively. These results indicate that the addition of microcapsules can enhance the frost resistance of the concrete, with CON3 exhibiting the most significant improvement. This is attributed to the formation of microcracks within the concrete after freeze–thaw cycles. The stress exerted during microcrack propagation can rupture the microcapsules and release the TDI, enabling the self-heal of the microcracks and enhancing the frost resistance of the concrete. Additionally, TM3 possesses the highest core score (72.6%) and the lowest 60-day weight loss (2.6%), implying a higher amount of healing agent available for healing microcracks. Therefore, the frost resistance of the concrete containing TM3 (CON3) is superior to that of the other concretes.

After 7 days of self-healing, the compressive strength recovery rates of CON0, CON1, CON2, and CON3 are 73.5%, 86.5%, 91.3%, and 96.9%, respectively (Table 7) [39]. Compared to CON0, the compressive strength recovery rates of CON1, CON2, and CON3 have increased by 17.7%, 24.2%, and 31.8%, respectively. The reason behind this is that microcracks develop within the concrete after freeze–thaw damage, and these microcracks encounter the microcapsules embedded within the concrete during expansion. Under the stress at the tip of the microcracks, the outer shell of the microcapsule ruptures, releasing TDI. TDI exhibits good fluidity and reacts rapidly with water in the surrounding environment, generating repair products to mend the microcracks and restore the mechanical properties of the concrete. Due to the higher initial core portion and better compactness of TM3, more TDI can be released to come into contact with water and participate in the self-healing reaction, forming a greater amount of repair products to fill the microcracks and restore the compressive strength of the concrete (CON3). Therefore, the compressive strength recovery rate of CON3 is higher than that of CON1 or CON2.

Table 8 reveals that the recovery rate of chloride diffusion coefficient after 7 days of self-healing is only 59.3% for CON0, whereas for CON1, CON2, and CON3, the recovery rates of chloride diffusion coefficient are 72.8%, 77.2%, and 84.6%, respectively [39]. Compared to CON0, the recovery rates of chloride diffusion coefficient for CON1, CON2, and CON3 have increased by 22.8%, 30.2%, and 42.7%, respectively. This is because microcracks develop within the concrete after freeze–thaw damage, and the stress at the tip of the microcracks causes the rupture of the microcapsule’s outer shell. The released TDI can react with water in the surrounding environment, rapidly generating repair products to mend the microcracks and improve the density of the concrete, thereby restoring its permeability. The recovery rate of chloride diffusion coefficient for CON3 is significantly higher than that of CON1 and CON2. This is attributed to the superior core portion, compactness, and micro-mechanical properties of TM3 compared to other microcapsules. After TM3 is ruptured by the stress at the tip of the microcracks, more TDI flows into the microcracks and reacts with water, forming repair products to fill the cracks and enhance the density of the concrete, leading to an improved recovery percentage of chloride diffusion coefficient (CON3).

#### 5.3.5. Sulfate Resistance and Self-Healing Ability of Concrete (Sulfate Dry–Wet Cycle)

**Figure 31:**
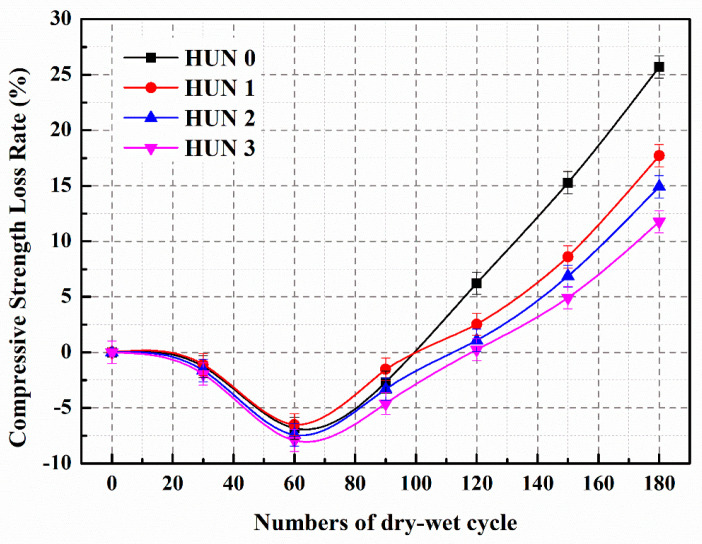
The compressive strength loss rate of concrete during dry–wet cycles [68], Copyright, 2021, Elsevier, License Number 5567390259421.

We prepared three types of microcapsules (Table 9) and added them into C30 concrete (Table 10). The sulfate resistance and self-healing ability of these concretes were studied through 180 cycles of sulfate dry–wet exposure tests [68]. Figure 31 illustrates the compressive strength loss rate of the concrete during the sulfate dry–wet cycles. The control concrete (HUN0) and the concrete containing microcapsules exhibited a negative growth in compressive strength loss rate during the first 90 dry–wet cycles. This negative growth in compressive strength loss rate is attributed to the infiltration of SO_4_^2−^ ions from the Na_2_SO_4_ solution into the specimens, reacting with hydration products to form expansive substances (gypsum and ettringite), resulting in volume expansion and a more compact internal structure of the concrete, thereby enhancing its compressive strength. During the dry–wet cycles, when the concrete specimens were removed and dried, there was minimal precipitation of sulfate crystals that filled the small pores within the concrete, leading to a more compacted structure and an improvement in the compressive strength of the specimens.

During the dry–wet cycles, the continuous infiltration of SO_4_^2−^ into the concrete leads to reactions with the matrix, resulting in the formation of gypsum and ettringite, which generates internal volumetric expansion pressure. Simultaneously, under drying conditions (the drying phase of the dry–wet cycle experiment), sulfate crystals precipitate within the concrete, causing crystallization pressure. The volume expansion and crystallization pressure within the concrete pores and inner walls increase with the number of dry–wet cycles. When the bearing capacity limit of the matrix is exceeded, microcracks develop internally, leading to a decrease in compressive strength. The appearance of microcracks accelerates the rate of SO_4_^2−^ ingress into the matrix, thereby hastening the sulfate corrosion of the concrete. Consequently, after 100 dry–wet cycles, the compressive strength loss rate of the concrete significantly increases. Following 180 dry–wet cycles, the compressive strength loss rates for HUN0, HUN1, HUN2, and HUN3 are 25.7%, 17.7%, 14.9%, and 11.8%, respectively. Figure 31 illustrates that the compressive strength loss rate of the concrete containing microcapsules is significantly lower than that of the control concrete after an equal number of dry–wet cycles. These results demonstrate that the addition of microcapsules in the concrete can enhance its sulfate resistance. This is because during the dry–wet cycles, sulfate erosion leads to the development of cracks within the concrete. The microcapsules in the concrete rupture under the stress of crack propagation, releasing IPDI, which reacts with moisture to form healing products, thereby mitigating the damage to the concrete structure and slowing down the decrease in compressive strength. Due to the superior mechanical properties, core content, and compaction degree of MS3 compared to MS1 and MS2, the internal structural damage of HUN3 is less severe, resulting in a lower compressive strength loss rate.

**Figure 32:**
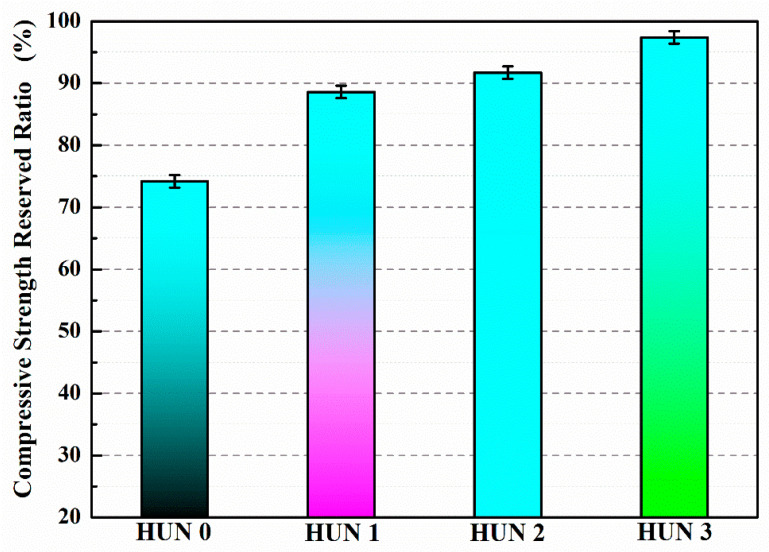
The compressive strength reserved ratio of concrete (180 dry–wet cycles) [68], Copyright, 2021, Elsevier, License Number 5567390259421.

Figure 32 displays the compressive strength reserved ratio of the concrete after 14 days of self-healing. The concrete containing microcapsules exhibits significantly higher compressive strength reserved ratio under sulfate erosion compared to HUN0 [68]. The compressive strength reserved ratio of HUN0 is 74.2%, while HUN1, HUN2, and HUN3 have compressive strength reserved ratio of 88.6%, 91.7%, and 97.4%, respectively. Compared to HUN0, HUN1, HUN2, and HUN3 show improvements of 19.4%, 23.6%, and 31.3% in compressive strength reserved ratio, respectively. The reason behind these results is that during the sulfate dry–wet cycles, SO_4_^2−^ from the Na_2_SO_4_ solution enters the concrete, reacting with hydration products to form gypsum and ettringite, leading to volume expansion of the concrete. The expansion stress within the concrete pores and inner walls gradually increases with the number of dry–wet cycles. When the expansion stress exceeds the bearing capacity limit of the matrix, microcracks appear in the concrete, accelerating the rate of SO_4_^2−^ ingress and rapidly increasing the rate of sulfate erosion. As a result, the compressive strength reserved ratio of the concrete decreases. However, when microcapsules are added to the concrete, the stress at the tip of the microcracks disrupts the shell of the microcapsules, releasing IPDI. IPDI rapidly reacts with water in the concrete, generating healing products that facilitate self-healing of the microcracks and restore their mechanical performance. The compressive strength reserved ratio of HUN3 is higher than that of HUN1 and HUN2. This is because MS3 possesses higher elastic modulus and hardness compared to MS1 and MS2, reducing the risk of fracture during concrete mixing. MS3 also has a higher initial core content, allowing more IPDI to react with water and form a greater amount of healing products in the concrete, ultimately resulting in an increased compressive strength reserved ratio.

**Figure 33:**
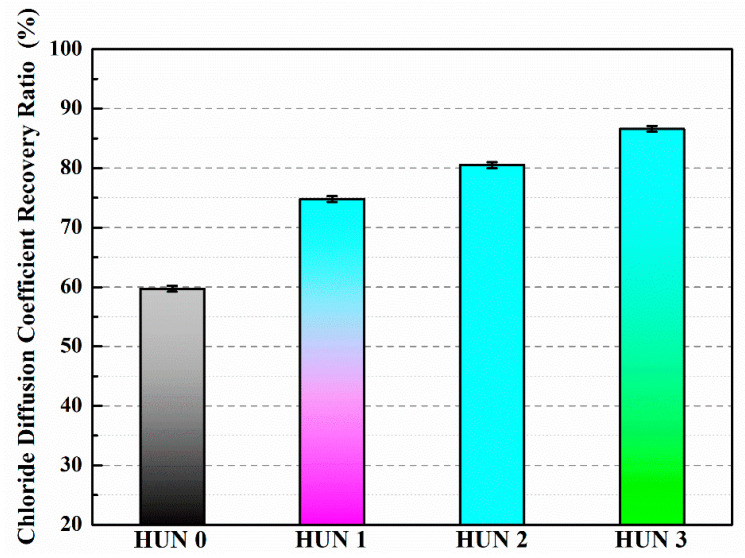
The chloride diffusion coefficient recovery ratio of concrete (180 dry–wet cycles) [68], Copyright, 2021, Elsevier, License Number 5567390259421.

Figure 33 illustrates the recovery ratio of chloride ion diffusion coefficients in the concrete after 14 days of self-healing (180 sulfate dry–wet cycles). The chloride diffusion coefficient recovery ratio for HUN0, HUN1, HUN2, and HUN3 are 59.7%, 74.8%, 80.5%, and 86.6%, respectively. The concrete containing microcapsules exhibits significantly higher recovery rates of chloride diffusion coefficients compared to HUN0, indicating excellent self-healing performance. This is because after 180 sulfate dry–wet cycles, several expansion products are formed within the concrete, resulting in the development of microcracks due to expansion stress. The stress at the tips of these microcracks causes the rupture of the microcapsule shells and the release of IPDI. The healing products generated from the reaction of IPDI with water fill the microcracks, enhancing the recovery ratio of chloride diffusion coefficients in the concrete containing microcapsules. The recovery ratio of chloride diffusion coefficient in HUN3 is higher than that in HUN1 and HUN2 because MS3 exhibits superior core content, compactness, and micro-mechanical properties compared to MS1 and MS2.

#### 5.3.6. Effect of Microcapsules on the Steel Corrosion in Cement-Based Materials

With the improvement in transportation networks, the requirements for the construction and protection of railways, highways, and bridges have also increased. Dong et al. [69] have developed a novel self-healing system for concrete materials using microcapsule technology, primarily aimed at corrosion protection of steel reinforcement. They characterized the performance of this system by analyzing the electrochemical impedance spectroscopy of steel reinforcement immersed in a simulated concrete environment. Experimental results demonstrate a strong inhibitory effect on corrosion caused by chloride ions when microcapsules are added at different mass fractions to a sodium chloride solution. A new equivalent circuit model is proposed, which takes into account the inductive effect of corrosion products formed on the steel surface, to explain the protective performance of microcapsules against steel reinforcement corrosion in concrete.

In order to effectively delay the corrosion of steel reinforcement in reinforced concrete transportation structures, Xu et al. [70] synthesized epoxy-urea–formaldehyde microcapsules with a size of 150–250 μm and prepared self-healing mortars with different microcapsule contents. The compressive strength and strength recovery rate were analyzed to select the appropriate amount of microcapsules for subsequent corrosion tests. Subsequently, two modes (without preloading/with preloading) were set up to simulate the original state and service state of the reinforced concrete structure. After undergoing accelerated corrosion through wet–dry cycles, electrochemical tests were conducted to analyze the corrosion performance of the embedded steel. The results showed that the addition of microcapsules slightly decreased the compressive strength of the self-healing mortar. At the same time, with the increase of microcapsule content, the strength recovery rate of the pre-damaged samples significantly improved. Finally, the study found that the addition of an appropriate amount of microcapsules can delay the time for steel reinforcement embedded in self-healing mortar to reach the chloride ion threshold. The anti-corrosion effect of embedded steel reinforcement in self-healing mortar is particularly significant. As the microcapsule content increases, the protection effect of steel reinforcement is enhanced.

In our previous work [39,53,57,60,68], we investigated the effects of mechanical rupture and electromagnetic-triggered release microcapsules on pore size distribution, mechanical properties, and surface crack self-healing of cement-based materials. Through experimental investigations, we found that the introduction of microcapsules had a positive impact on the pore size distribution, mechanical properties, impermeability, and surface cracks self-healing of damaged cement-based materials. Additionally, we observed that electromagnetic-triggered release microcapsules exhibited superior self-healing ability compared to mechanical fracture ruptured microcapsules. In addition, adding an appropriate amount of microcapsules can delay the time when the steel bars are embedded in the mortar and reach the chloride ion threshold. The anti-corrosion effect of embedded steel bars in the mortar is particularly evident. As the content of microcapsules increases, the protective effect of steel bars increases.

## 6. Future Development Prospects

This article provides a comprehensive review and analysis of the achievements in the field of cement-based material with microcapsule self-heal, based on existing research. It also explores future research directions in the field of microcapsule self-heal technology. The summary is as follows:

(1) Optimization of microcapsule design: future research can focus on optimizing the design of microcapsules, including the selection of core materials, improvement of shell materials, and control of microcapsule size and distribution. By designing the structure and properties of microcapsules in a rational manner, the effectiveness and durability of self-healing materials can be further improved.
(2) Enhancement of microcapsule self-healing ability: further research can delve into the self-healing mechanisms of microcapsules, such as introducing new self-healing components or modifying the internal structure of microcapsules to enhance their self-healing efficiency and capability. Additionally, the combination of microcapsules with other functional materials can be investigated to achieve multifunctional self-healing effects.
(3) Controlled release of microcapsules: researchers can explore more precise mechanisms for the release of microcapsules, such as electromagnetic, chemical, or biological stimuli, to control the release behavior of microcapsules. This will help achieve precise control and regulation of the self-healing process, thereby improving the performance and applicability of self-healing materials.
(4) Material performance evaluation and standardization: future research can focus on developing comprehensive evaluation methods and standards to assess the performance and durability of microcapsule self-heal materials. Establishing unified testing methods and evaluation criteria will promote the development of the field and facilitate the practical application and industrialization of self-healing materials.
(5) Application expansion and engineering practice: further application of microcapsule self-heal technology in practical engineering projects and conducting long-term usage and environmental adaptation studies are encouraged. Through experiments and monitoring under different environmental and load conditions, the feasibility and reliability of microcapsule self-heal materials can be verified, promoting their practical application in engineering.

In summary, future research can focus on the optimization of microcapsule self-heal material design, exploration of self-healing mechanisms, control of release behavior, performance evaluation, and application expansion. These studies will provide references for related research on cement-based materials with microcapsule self-heal and promote the development of this field.

## Figures and Tables

**Table 1:** Particle sizes of microcapsules [57].

Microcapsule	D10 Values/μm	D50 Values/μm	D90 Values/μm
WM1	10	23	50
WM2	25	52	115

**Table 2:** Elastic modulus and hardness of microcapsules [57].

Microcapsules	Elastic Modulus (GPa)	Hardness (MPa)
WM1	0.55	4.89
WM2	2.02	72.54

**Table 3:** Flexural strength and compressive strength of mortars [53].

Mortars *	Flexural Strength	Compressive Strength
SJ-0	8.2 MPa	33.4 MPa
SJ-1	7.9 MPa	32.6 MPa
SJ-2	7.5 MPa	30.9 MPa

* SJ-0: control mortar; * SJ-1: the mortar containing SWMs; * SJ-2: the mortar containing DWMs.

**Table 4:** Compressive strength of mortars [57].

Mortars *	Compressive Strength (after 28 Days of Standard Curing)
CCM0	31.2 MPa
CCM1	40.6 MPa
CCM2	35.5 MPa

* CCM0: control mortar; * CCM1: the mortar containing WM1; * CCM2: the mortar containing WM2.

**Table 5:** Specific process parameters and basic properties of microcapsules [39], Copyright, 2021, Elsevier, License Number 5567380935164.

	TM1 *	TM2 *	TM3 *
**Mass Ratio of Raw Materials (%)**	Paraffin 33 TDI 67	Paraffin 16.5 PE Wax 16.5 TDI 67	Paraffin 15 PE Wax 15 Nano-SiO_2_ 3 TDI 67
**Preparation Temperature (°C)**	75	120	120
**Agitation Rate (rpm)**	600	800	800
**Softening Point (°C)**	58	95	100
**Average Particle Size (μm)**	100	320	480
**Core Fraction (%)**	66.5	68.8	72.6
**Elastic Modulus (GPa)**	0.48	0.83	1.87
**Hardness (MPa)**	4.06	5.90	61.67
**Weight Loss Rate in 60 d (%)**	13.5	7.2	2.6

* TM1: paraffin encapsulated TDI microcapsule, **TM2:** paraffin/PE wax encapsulated TDI microcapsule, **TM3:** nano-SiO_2_/paraffin/PE wax encapsulated TDI microcapsule.

**Table 6:** Mix designs of concrete containing microcapsules by mass ratio (kg/m^3^) [39], Copyright, 2021, Elsevier, License Number 5567380935164.

Specimens	Cement	River Sand	Gravel	FA	Water	Superplasticizer	Microcapsules
**CON0**	280	850	1080	60	160	5.5	0
**CON1**	280	850	1080	60	160	5.5	8.4 (TM1)
**CON2**	280	850	1080	60	160	5.5	8.4 (TM2)
**CON3**	280	850	1080	60	160	5.5	8.4 (TM3)

**Table 7:** The compressive strength recovery rates of concretes [39], Copyright, 2021, Elsevier, License Number 5567380935164.

Specimens	CON0	CON1	CON2	CON3
**Compressive Strength Recovery Rate (%)**	73.5	86.5	91.3	96.9

**Table 8:** The chloride diffusion coefficient recovery rate of concrete [39], Copyright, 2021, Elsevier, License Number 5567380935164.

Specimens	CON0	CON1	CON2	CON3
**Chloride Diffusion Coefficient Recovery Rate (%)**	59.3	72.8	77.2	84.6

**Table 9:** Specific process parameters and basic properties of microcapsules [68], Copyright, 2021, Elsevier, License Number 5567390259421.

	MS1 *	MS2 *	MS3 *
**Mass Ratio of Raw Materials (%)**	Paraffin Wax 33 IPDI 67	Paraffin Wax 16.5 Polyethylene Wax 16.5 IPDI 67	Paraffin Wax 15 Polyethylene Wax 15 Nano Silica 3 IPDI 67
**Temperature (°C)**	80	130	140
**Stirring Speed (rpm)**	800	900	1000
**Average Particle Size (μm)**	90	300	500
**Core Content (%)**	69.7	73.5	77.8
**Elastic Modulus (GPa)**	0.52	0.96	2.01
**Hardness (MPa)**	4.33	6.12	65.99
**Weight Loss Rate in 60 Days (%)**	12.1	6.5	1.9

MS1 *: paraffin wax encapsulated IPDI microcapsule; MS2 *: paraffin wax/polyethylene wax encapsulated IPDI microcapsule; MS3 *: nano silica/paraffin wax/polyethylene wax encapsulated IPDI microcapsule.

**Table 10:** Mix design of concrete containing microcapsules by mass ratio (kg/m^3^) [68], Copyright, 2021, Elsevier, License Number 5567390259421.

Constituent	HUN0	HUN1	HUN2	HUN3
**Cement**	260	260	260	260
**Mineral Powder**	120	120	120	120
**Fly Ash**	140	140	140	140
**Sand**	816	816	816	816
**Crushed Stone**	850	850	850	850
**Water**	180	180	180	180
**Superplasticizer**	13	13	13	13
**Microcapsules**	0	8.4 (MS1)	8.4 (MS2)	8.4 (MS3)

## References

[1] Tittelboom K.V., De Belie N. (2013). Self-healing in cementitious materials—A review. Materials.

[2] Meharie M.G., Kaluli J.W., Abiero-Gariy Z., Kumar N.D. (2017). Factors Affecting the Self-Healing Efficiency of Cracked Concrete Structures. Am. J. Appl. Sci. Res..

[3] Aïtcin P.-C. (2000). Cements of yesterday and today: Concrete of tomorrow. Cem. Concr. Res..

[4] Mehta P.K. (1999). Concrete technology for sustainable development. Concr. Int..

[5] Maddalena R., Bonanno L., Balzano B., Tuinea-Bobe C., Sweeney J., Mihai I. (2020). A crack closure system for cementitious composite materials using knotted shape memory polymer (k-SMP) fibres. Cem. Concr. Compos..

[6] Ismail M., Toumi A., François R., Gagné R. (2008). Effect of crack opening on the local diffusion of chloride in cracked mortar samples. Cem. Concr. Res..

[7] Picandet V., Khelidj A., Bellegou H. (2009). Crack effects on gas and water permeability of concretes. Cem. Concr. Res..

[8] Hoseini M., Bindiganavile V., Banthia N. (2009). The effect of mechanical stress on permeability of concrete: A review. Cem. Concr. Compos..

[9] Dry C. (1994). Matrix cracking repair and filling using active and passive modes for smart timed release of chemicals from fibers into cement matrices. Smart Mater. Struct..

[10] Carpinteri A. (1990). Size-Scale Effects on the Brittleness of Concrete Structures: Dimensional Analysis and Snap-Back Instability. Spec. Publ..

[11] Sidiq A., Gravina R., Giustozzi F. (2019). Is concrete healing really efficient? A review. Constr. Build. Mater..

[12] Li W., Liu W., Wang S. (2017). The Effect of Crack Width on Chloride-Induced Corrosion of Steel in Concrete. Adv. Mater. Sci. Eng..

[13] Sideris K.K., Anagnostopoulos N.S. (2013). Durability of normal strength self-compacting concretes and their impact on service life of reinforced concrete structures. Constr. Build. Mater..

[14] Li V.C., Herbert E. (2012). Robust Self-Healing Concrete for Sustainable Infrastructure. J. Adv. Concr. Technol..

[15] Trask R., Williams H., Bond I. (2007). Self-repairing polymer composites: Mimicking nature to enhance performance. Bioinspiration Biomim..

[16] Van Belleghem B., Van Tittelboom K., De Belie N. (2018). Efficiency of self-healing cementitious materials with encapsulated polyurethane to reduce water ingress through cracks. Mater. Des. Constr..

[17] Pattanaik S.C. (2011). Causes, Evaluation and repair of cracks in concrete structures. ACI Mater. J..

[18] Emmons P.H., Sordyl D.J. (2006). The state of the concrete repair industry, and a vision for its future. Concr. Repair Bull..

[19] Edvardsen C. (1999). Water permeability and autogenous healing of cracks in concrete. ACI Mater. J..

[20] De Muynck W., De Belie N., Verstraete W. (2010). Microbial carbonate precipitation in construction materials: A review. Ecol. Eng..

[21] Jonkers H.M., Thijssen A., Muyzer G., Copuroglu O., Schlangen E. (2010). Application of bacteria as self-healing agent for the development of sustainable concrete. Ecol. Eng..

[22] Stocks-Fischer S., Galinat J.K., Bang S.S. (1999). Microbiological precipitation of CaCO_3_. Soil Biol. Biochem..

[23] Wiktor V., Jonkers H.M. (2011). Quantification of crack-healing in novel bacteria-based self-healing concrete. Cem. Concr. Compos..

[24] Issa M.A., Tsui S., Yousif A. (1995). Application of knowledge-based expert systems for rating highway bridges. Eng. Fract. Mech..

[25] Roig-Flores M., Pirritano F., Serna P., Ferrara L. (2016). Effect of crystalline admixtures on the self-healing capability of early-age concrete studied by means of permeability and crack closing tests. Constr. Build. Mater..

[26] Van Tittelboom K., De Belie N., Van Loo D., Jacobs P. (2011). Self-healing efficiency of cementitious materials containing tubular capsules filled with healing agent. Cem. Concr. Compos..

[27] Ferrara L., Krelani V., Carsana M. (2014). A “fracture testing” based approach to assess crack healing of concrete with and without crystalline admixtures. Constr. Build. Mater..

[28] Hearn N. (1998). Self-sealing, autogenous healing and continued hydration: What is the difference?. Mater. Struct..

[29] Huang H., Ye G., Qian C., Schlangen E. (2016). Self-healing in cementitious materials: Materials, methods and service conditions. Mater. Des..

[30] De Belie N., Gruyaert E., Al-Tabbaa A., Antonaci P., Baera C., Bajare D., Darquennes A., Davies R., Ferrara L., Jefferson T. (2018). A Review of Self-Healing Concrete for Damage Management of Structures. Adv. Mater. Interfaces.

[31] Gupta S., Pang S.D., Kua H.W. (2017). Autonomous healing in concrete by bio-based healing agents—A review. Constr. Build. Mater..

[32] Sisomphon K., Copuroglu O., Koenders E. (2012). Self-healing of surface cracks in mortars with expansive additive and crystalline additive. Cem. Concr. Compos..

[33] Nathaniel O., Sam A.R.M., Lim N.H.A.S., Adebisi O., Abdulkareem M. (2020). Biogenic approach for concrete durability and sustainability using effective microorganisms: A review. Constr. Build. Mater..

[34] Norris C.J., Bond I.P., Trask R.S. (2011). Interactions between propagating cracks and bioinspired self-healing vascular embedded in glass fiber reinforced composites. Compos. Sci. Technol..

[35] Han T., Wang X., Li D., Li D., Xing F., Han N., Li Z. (2021). Uniaxial deformation characteristics and mechanical model of microcapsule-based self-healing cementitious composite. Constr. Build. Mater..

[36] Wang X., Zhang J., Han R., Han N., Xing F. (2019). Evaluation of damage and repair rate of self-healing microcapsule-based cementitious materials using electrochemical impedance spectroscopy. J. Clean. Prod..

[37] Xu N., Song Z., Guo M.-Z., Jiang L., Chu H., Pei C., Yu P., Liu Q., Li Z. (2021). Employing ultrasonic wave as a novel trigger of microcapsule self-healing cementitious materials. Cem. Concr. Compos..

[38] Du W., Liu Q., Lin R., Yu J. (2021). Influence of external environment on self-repairing ability of the cement-based materials containing paraffin/toluene-di-isocyanate microcapsules. Constr. Build. Mater..

[39] Du W., Liu Q., Lin R. (2021). Effects of toluene-di-isocyanate microcapsules on the frost resistance and self-repairing capability of concrete under freeze-thaw cycles. J. Build. Eng..

[40] Tsuda N., Ohtsubo T., Fuji M. (2012). Preparation of self-bursting microcapsules by interfacial polymerization. Adv. Powder Technol..

[41] Yang J., Keller M.W., Moore J.S., White S.R., Sottos N.R. (2008). Microencapsulation of Isocyanates for Self-Healing Polymers. Macromolecules.

[42] Salaün F., Devaux E., Bourbigot S., Rumeau P. (2009). Influence of process parameters on microcapsules loaded with n-hexadecane prepared by in situ polymerization. Chem. Eng. J..

[43] Huang Q., Gong S., Han W., Chen Y., Shu X. (2020). Preparation of TTO/UF resin microcapsule via in situ polymerisation and modelling of its slow release. J. Microencapsul..

[44] Muhoza B., Yuyang H., Uriho A., Harindintwali J.D., Qian L., Li Y. (2023). Spray-and freeze-drying of microcapsules prepared by complex coacervation method: A review. Food Hydrocoll..

[45] Liu Y.Q., Ding W.J., Dong P., Han S.W., Li H.F., Chen S.S., Hong S.X., Dong B.Q., Xing F. (2017). Preparation and anti-corrosion performances of chemical self-healing system for steel in concrete. J. Funct. Mater..

[46] Du W., Yu J., Gu Y., Li Y., Zhang C., Liu Q. (2019). Preparation and application of microcapsules containing toluene-di-isocyanate for self-healing of concrete. Constr. Build. Mater..

[47] Li Y., Yu J., Cao Z., He P., Liu Q., Han X., Wan Y. (2021). Preparation and application of novel microcapsules ruptured by microwave for self-healing concrete. Constr. Build. Mater..

[48] Xue B., Wang H., Pei J., Li R., Zhang J., Fan Z. (2017). Study on self-healing microcapsule containing rejuvenator for asphalt. Constr. Build. Mater..

[49] Tao Y., Yan X. (2022). Influence of HLB Value of Emulsifier on the Properties of Microcapsules and Self-Healing Properties of Waterborne Coatings. Polymers.

[50] Zhao W., Yan X. (2022). Preparation of Thermochromic Microcapsules of Bisphenol A and Crystal Violet Lactone and Their Effect on Coating Properties. Polymers.

[51] Tian Y., Liu Y., Zhang L., Hua Q., Liu L., Wang B., Tang J. (2020). Preparation and characterization of gelatin-sodium alginate/paraffin phase change microcapsules. Colloids Surfaces A Physicochem. Eng. Asp..

[52] Du W., Yu J., He B., He Y., He P., Li Y., Liu Q. (2020). Preparation and characterization of nano-SiO_2_/paraffin/PE wax composite shell microcapsules containing TDI for self-healing of cementitious materials. Constr. Build. Mater..

[53] Li E., Du W., Zhuang R., Ba M., Yuan L., Zhang Q., Zhang Y. (2022). Preparation and Characterization of Electromagnetic-Induced Rupture Microcapsules for Self-Repairing Mortars. Materials.

[54] Xu D., Chen W., Fan X. (2020). Experimental investigation of particle size effect on the self-healing performance of microcapsule for cemented coral sand. Constr. Build. Mater..

[55] Yao J.L., Yang C.P., Zhu C.F., Hou B.Q. (2019). Preparation process of epoxy resin microcapsules for self-healing coatings. Prog. Org. Coat..

[56] Zotiadis C., Patrikalos I., Loukaidou V., Korres D.M., Karantonis A., Vouyiouka S. (2021). Self-healing coatings based on poly(urea-formaldehyde) microcapsules: In situ polymerization, capsule properties and application. Prog. Org. Coat..

[57] Du W., Li E.W., Lin R.S. (2022). Preparation and characterization of nano-CaCO_3_/ceresine wax composite shell microcapsules containing E-44 epoxy resin for self-healing of cement-based materials. Nanomaterials.

[58] Zhang B., Li S., Fei X., Zhao H., Lou X. (2020). Enhanced mechanical properties and thermal conductivity of paraffin microcapsules shelled by hydrophobic-silicon carbide modified melamine-formaldehyde resin. Colloids Surfaces A Physicochem. Eng. Asp..

[59] Li M., Chen M., Wu Z. (2014). Enhancement in thermal property and mechanical property of phase change microcapsule with modified carbon nanotube. Appl. Energy.

[60] Du W., Liu Q., Lin R., Su X. (2021). Preparation and Characterization of Microcrystalline Wax/Epoxy Resin Microcapsules for Self-Healing of Cementitious Materials. Materials.

[61] Ji X., Wang S., Yao B., Si W., Wang C., Wu T., Zhang X. (2023). Preparation and properties of nano-SiO_2_ modified microcapsules for asphalt pavement. Mater. Des..

[62] Ahangaran F., Hayaty M., Navarchian A.H., Picchioni F. (2017). Micromechanical assessment of PMMA microcapsules containing epoxy and mercaptan as self-healing agents. Polym. Test..

[63] White S.R., Sottos N.R., Geubelle P.H., Moore J.S., Kessler M.R., Sriram S.R., Brown E.N., Viswanathan S. (2001). Autonomic healing of polymer composites. Nature.

[64] Ryu S.A., Hwang Y.-H., Oh H., Jeon K., Lee J.H., Yoon J., Lee J.B., Lee H. (2021). Biocompatible Wax-Based Microcapsules with Hermetic Sealing for Thermally Triggered Release of Actives. ACS Appl. Mater. Interfaces.

[65] Tylkowski B., Giamberini M., Underiner T., Prieto S.F., Smets J. (2016). Photo-Triggered Microcapsules. Macromol. Symp..

[66] Wang H.C., Grolman J.M., Rizvi A., Hisao G.S., Rienstra C.M., Zimmerman S.C. (2017). pH-triggered release from polyamide microcapsules prepared by interfacial polymerization of a simple diester monomer. ACS Macro Lett..

[67] Xiong W., Tang J., Zhu G., Han N., Schlangen E., Dong B., Wang X., Xing F. (2015). A novel capsule-based self-recovery system with a chloride ion trigger. Sci. Rep..

[68] Du W., Lin R., Liu Q. (2021). Investigation of isophorone diisocyanate microcapsules to improve self-healing properties and sulfate resistance of concrete. Constr. Build. Mater..

[69] Dong B., Wang Y., Ding W., Li S., Han N., Xing F., Lu Y. (2014). Electrochemical impedance study on steel corrosion in the simulated concrete system with a novel self-healing microcapsule. Constr. Build. Mater..

[70] Xu N., Ji X.P., Wang W.Y. (2020). Effect of epoxy/urea-formaldehyde microcapsules on corrosion performance of reinforcing steel in cement mortars. J. Corros. Sci. Eng..

